# Protoporphyrin IX Stimulates Melanogenesis, Melanocyte Dendricity, and Melanosome Transport Through the cGMP/PKG Pathway

**DOI:** 10.3389/fphar.2020.569368

**Published:** 2020-09-11

**Authors:** Jinpeng Lv, Xiaohong An, Songzhou Jiang, Ying Yang, Guoqiang Song, Rongyin Gao

**Affiliations:** ^1^ Department of Pharmacy, College of Pharmaceutical Engineering and Life Sciences, Changzhou University, Changzhou, China; ^2^ Shanghai Jiyan Bio-pharmaceutical Co., Ltd., Shanghai, China; ^3^ Department of Pharmacy, The First People’s Hospital of Changzhou, The Third Affiliated Hospital of Soochow University, Changzhou, China

**Keywords:** protoporphyrin IX, melanogenesis, melanosome transport, guanylate cyclase, cyclic guanosine 3’, 5’-monophosphate/protein kinase G

## Abstract

Protoporphyrin IX (PPIX) is a heterocyclic organic compound that is the last intermediate in the heme biosynthetic pathway. PPIX, due to its photodynamic effects, is utilized in the treatment of skin diseases. Furthermore, PPIX has been utilized as a melanogenesis-stimulating agent in various studies. However, the exact function and mechanism underlying PPIX action in melanocytes remain to be elucidated. In the present study, we sought to further investigate how PPIX affects melanocyte melanogenesis, and whether PPIX is involved in melanin transport. Our findings demonstrated that PPIX increased melanocyte dendricity and melanosome transport, in addition to increasing melanogenesis. PPIX functions primarily by activating the guanylate cyclase (GC) and cyclic guanosine 3’, 5’-monophosphate/protein kinase G (cGMP/PKG) signaling pathways. Once activated, these pathways increase tyrosinase activity and the expression of microphthalmia-associated transcription factor (MITF), tyrosinase, tyrosinase-related protein-1 and -2 (TRP-1 and TRP-2), myosin Va, melanophinin, Ras-related protein Rab-27A (Rab27a), and cell division cycle 42 (Cdc42), promoting melanogenesis, melanocyte dendricity, and melanosome transport. Furthermore, the melanogenic effects of PPIX were confirmed *in vivo* in a zebrafish model system. Our results indicate that PPIX is not cytotoxic and may, thus, be utilized as a pigmentation enhancer.

## Introduction

In humans, melanin originating from melanocytes plays a variety of valuable physiological functions, with the most important being the protection of the human skin against damage from ultraviolet (UV) radiation (Raposo and Marks, 2007; Noguchi et al., 2014). Melanin biosynthesis occurs in melanocytes, and its storage is restricted to melanosomes, which are melanocyte-specific lysosome-related organelles (Marks and Seabra, 2001). The mature melanosomes migrate along the microtubules and actin filaments from the perinuclear area to the cell membrane and are eventually transferred to neighboring keratinocytes (Beaumont et al., 2011; Wu et al., 2018; Makbal et al., 2020).

Several intrinsic and extrinsic factors involved in skin pigmentation have been identified. The key enzyme that regulates melanogenesis is tyrosinase, and it acts with tyrosinase-related protein 1 (TRP-1) and tyrosinase-related protein 2 (TRP-2) to promote melanogenesis (Park et al., 2020). Microphthalmia-associated transcription factor (MITF), a key activator of the tyrosinase promoter, is a master transcription factor that activates the transcription of pigmentation genes and is required for melanocyte proliferation and survival (Lee et al., 2013; Lv et al., 2020), whereas kinesin family member 5b (KIF5b) functions as a motor that regulates outward melanosome transport along microtubules (Hara et al., 2000; Hirokawa et al., 2009). >>myosin Va-melanophilin-Ras–related protein Rab-27A (Rab27a) complexes contribute to melanosome transport along actin filaments, while Rab27A-melanophinin complex is responsible for anchoring melanosomes transferred to the cell membrane (Ohbayashi and Fukuda, 2012). Furthermore, cell division cycle 42 (Cdc42) contributes to dendrite extension, a crucial requirement for melanosome transport (Luo, 2000). Environmental stimuli, such as UV irradiation, play an important role in melanogenesis and dendrite extension (Abdel-Naser et al., 2003). Upon exposure to UV radiation, activated keratinocytes and melanocytes produce α-melanocyte–stimulating hormone (α-MSH) (D’Mello et al., 2016). When α-MSH binds to melanocortin-1 receptor (MC1R) in melanocytes, it can activate adenylate cyclase (AC), increase the intracellular cyclic adenosine monophosphate (cAMP) levels, and subsequently activate the protein kinase A (PKA)/cAMP-responsive element-binding (CREB) pathway, promoting melanogenesis (Corre et al., 2004; Rzepka et al., 2016). In addition, UV radiation directly activates guanylate cyclase (GC), increases the intracellular levels of cyclic guanosine monophosphate (cGMP), and subsequently activates the protein kinase G (PKG)/CREB pathway, finally promoting melanogenesis (Romero-Graillet et al., 1996).

Protoporphyrin IX (PPIX) is a heterocyclic organic compound that consists of four pyrrole rings and is the immediate precursor to heme (Sachar et al., 2016). Accumulation of PPIX produces photosensitivity and skin damage (Thapar and Bonkovsky, 2008; Dailey and Meissner, 2013). Due to its fluorescence properties, PPIX has been used clinically for the treatment of rhinophyma, sebaceous gland hyperplasia, photo rejuvenation, and cosmetic enhancement (Sachar et al., 2016). Previous studies have suggested that PPIX markedly activates soluble GC purified from bovine lung (Ignarro et al., 1984) and that PPIX promotes melanogenesis in melanoma cells (Kim et al., 2006; Alesiani et al., 2009; Kim et al., 2016). However, the exact function and mechanism underlying PPIX action in melanocytes remain to be elucidated. Therefore, the *in vitro* and *in vivo* effects of PPIX on pigmentation and the associated mechanisms require further study.

This study provides the first extensive description of the exact function of PPIX in skin pigmentation. Specifically, our results suggest that PPIX not only participates in melanin biosynthesis but also promotes melanosome transport. These functions can be ascribed to the activation of the GC/cGMP/PKG signaling pathway. Once this pathway was activated, it increased the expression of MITF, tyrosinase, myosin Va, melanophinin, Rab27a, and Cdc42, finally increasing the pigmentation. In the *in vivo* experiments, PPIX increased tyrosinase activity and body pigmentation in zebrafish.

## Materials and Methods

### Materials

Protoporphyrin IX (CAS: 553-12-8; purity, >95%) and tyrosinase derived from mushrooms (T128536) were purchased from Aladdin (Shanghai, China). Antibodies against tyrosinase (ab180753, 1:500), MITF (ab20663, 1:2,000), TRP-1 (ab3312, 1:1,000), TRP-2 (ab221144, 1:1,000), and cytokeratin (ab7753, 1:200) were purchased from Abcam (Cambridge, UK). p-CREB (9198S, 1:1,000) and the antibody against CREB (9197S, 1:1000) were obtained from Cell Signaling Technology (MA, USA). LY83583 (sc-200314A), KT5823 (sc-3534B), and antibodies against GP100 (sc-393094, 1:500), KIF5b (sc-133184, 1:500), myosin Va (sc-365986, 1:500), melanophinin (sc-365735, 1: 500), Rab27a (sc-74586, 1: 500), and Cdc42 (sc-8401, 1:500) were obtained from Santa Cruz Biotechnology (CA, USA). RT-qPCR kits were purchased from Takara Biomedical Technology (Beijing, China). The BCA protein assay kit (P0011), cell lysis buffer (P0013), cGMP assay kit, antibody against β-actin (AF0003), donkey anti-rabbit immunoglobulin G (IgG) (Alexa Fluor 555–labeled) (A0453, 1:500), and goat anti-mouse IgG (Alexa Fluor 488–labeled) (A0428, 1:500) were obtained from Beyotime Biotechnology (Shanghai, China).

### Cell Culture

SK-MEL-2 and HaCaT cells were purchased from the Cell Bank, Chinese Academy of Sciences. The cells were cultured in Dulbecco’s modified Eagle’s medium (DMEM) (Gibco, USA) supplemented with 10% fetal bovine serum (FBS) (HyClone, USA) at 37°C and 5% CO_2_. Human epidermal melanocytes (HNM) were obtained from Sciencell Research Laboratories (CA, USA) and incubated in 254CF medium (Gibco, USA) containing human melanocyte growth supplement at 37°C and 5% CO_2_ (Lv et al., 2019).

### Cell Viability Assay

Cell viability was measured using the 3-(4,5-dimethylthiazol-2-yl)-2,5-diphenyltetrazolium bromide (MTT) assay. Briefly, cells were seeded at a density of 2,500 cells/well in 96-well plates. After 24 h, the cells were treated with different concentrations of PPIX, and after 48 h, the cells were incubated with 20 μL of MTT working solution for another 4 h. After removing the solution, dimethyl sulfoxide (DMSO) (200 μL) was added to each well, and the absorbance was measured at 570 nm using a microplate spectrophotometer (BioTek Instruments).

### Measurement of Melanin Content

The melanin content was measured as previously described (Lv et al., 2015; Lv et al., 2020). Briefly, the total melanin in the cell plate was dissolved in 100 μL of NaOH working solution (1 mol/L, 10% DMSO) at 80°C for 2 h, and the melanin content was estimated by measuring the absorbance at 405 nm.

### Tyrosinase Activity

Cellular tyrosinase activity was examined according to a previously described procedure (Kim et al., 2008; Zhou et al., 2016). Briefly, the cells were seeded at a density of 1× 10^5^ cells/well in a six-well plate. After 24 h, the cells were treated with the indicated concentrations of PPIX for 48 h and then lysed using cell lysis buffer. The lysates were clarified by centrifugation at 12,000 rpm for 20 min at 4°C. After protein quantification, 100 μL of phosphate buffered saline (PBS) (0.1 M, pH 6.5) containing 30 μg of protein was mixed with 100 μL of levodopa (L-DOPA) (0.1%). Following incubation at 37°C for 1 h, the optical absorbance was examined at 475 nm. A cell-free assay system was used to examine the direct effects of PPIX on tyrosinase activity (Kim et al., 2008; Lee et al., 2013). In brief, 100 μL of PBS (0.1 M, pH 6.5) containing the indicated concentrations of PPIX was mixed with 10 units of mushroom tyrosinase and 50 μL of L-tyrosine (0.03%), and the mixture was incubated at 37°C for 10 min. Then, absorbance was measured at 475 nm.

### Fontana-Masson Staining

Melanoma cells (SK-MEL-2) were fixed in 4% paraformaldehyde for 20 min and then rinsed three times with ddH_2_O. The fixed cell samples were subsequently stained with Fontana ammoniacal silver solution overnight at 25°C. After washing three times with ddH_2_O, the cells were stained with Hypo solution for 3 min, rinsed with ddH_2_O, and counterstained with neutral red stain for 5 min. Finally, the cells were rinsed with ddH_2_O, dehydrated in 100% ethanol, and mounted on slides for observation under a microscope (Gu et al., 2018).

### Melanosome Transport Assays

HaCaT and SK-MEL-2 cells were cultured on a confocal dish following a keratinocyte and melanocyte co-culture protocol (Lee et al., 2015). The cells were immunostained with anti-cytokeratin and anti-GP100 according to the standard immunofluorescence protocol after treatment with PPIX. Images were obtained using an Olympus BX41 fluorescent microscope (Tokyo, Japan). The transfer of melanosome from SK-MEL-2 to HaCaT was quantified (Singh et al., 2017). Briefly, evaluation of melanosome transfer in co-cultured SK-MEL-2 and HaCaT cells was performed by counting fluorescent GP100-positive spots within recipient HaCaT cells in five random microscopic fields per well at 400× magnification in three independent experiments.

### Reverse Transcription–Quantitative PCR (RT-qPCR)

SK-MEL-2 cells and HNM total RNA was extracted using TRIzol reagent and quantified spectrophotometrically. Single-stranded cDNA was synthesized using SuperScript II Reverse Transcriptase according to the manufacturer’s instructions. SYBR-Green quantitative PCR analysis was performed with an ABI PRISM Sequence Detection System (Applied Biosystems) and pre-validated primer sets. All samples were run in triplicate. Threshold cycles were placed in the logarithmic portion of the amplification curve, and the results were normalized to GAPDH. The fold difference between two samples was determined by the delta-delta Cq method (Lv et al., 2020). The primer sequences are presented in **Supplement Materials Table I and II**.

### Western Blot Analysis

The protein samples (30 μg protein/sample) were separated using 10% sodium dodecyl sulfate polyacrylamide gel electrophoresis (SDS PAGE) and then transferred onto a nitrocellulose filter (NC) membrane using an electrophoretic transfer system (Bio-Rad, USA). Subsequently, the membranes were blocked with blocking buffer containing 2.5% bovine serum albumin (BSA) in Tris-buffered saline and 0.1% Tween 20 (TBST) for 1.5 h at 25°C and incubated with the corresponding primary antibodies overnight at 4°C. Next, the blots were incubated with peroxidase-conjugated secondary antibodies for 1 h at 25°C and visualized using enhanced chemiluminescence (Liao et al., 2017; Yun et al., 2020). The western blot results shown here are representative of three experiments.

### Enzyme-Linked Immunosorbent Assay

Melanoma cells (SK-MEL-2) or human melanocytes were treated with PPIX for 1 h. The cells were lysed with Triton X-100 (1%) at 4°C for 1 h, and the lysates were clarified by centrifugation at 12,000 rpm for 20 min at 4°C. The supernatant was used to assess the level of cGMP and the activities of GC and PKG using ELISA assay kits (IBL, Hamburg, Germany), following the manufacturer’ s instructions.

### Phenotype-Based Evaluation and Tyrosinase Activity Assays in Zebrafish

Phenotype-based evaluation was conducted according to a previous study (Choi et al., 2007; Agalou et al., 2018). Synchronized embryos were collected and arrayed in 96-well plates (three to four embryos per well with 200-μL medium). PPIX dissolved in DMSO (0.1%) was added to the embryo medium for 35 to 60 h. The effects of PPIX on the pigmentation of zebrafish were detected using an Olympus SZX2 stereomicroscope (Tokyo, Japan). Tyrosinase activity was measured according to a previous study (Choi et al., 2007). A total of 50 zebrafish embryos were lysed using cell lysis buffer, and the lysates were clarified by centrifugation at 12,000 rpm for 20 min at 4°C. After protein quantification, 100 μg of total protein was added to 50 μL of 1 mM L-DOPA, and absorbance was measured at 475 nm following incubation with L-DOPA for 60 min at 37°C.

### Statistical Analysis

All the experiments were performed in triplicate, and the results were expressed as the mean ± standard error of the mean (SEM). Statistical analysis was performed using one-way ANOVA following Turkey’s *post hoc* test for multiple comparison tests using GraphPad Prism 5.0 software. A P value of <0.05 was considered statistically significant.

## Results

### PPIX Induced Melanogenesis

**Figure 1:**
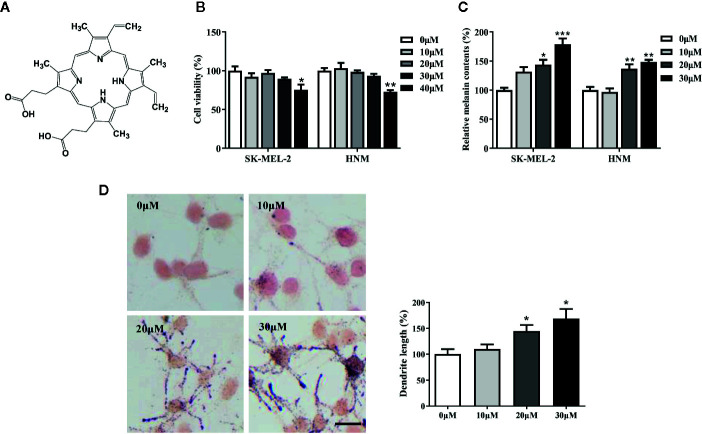
Protoporphyrin IX (PPIX) induced hyperpigmentation in melanocytes. **(A)** The chemical structure of PPIX. **(B)** After incubation of with various concentrations (10-40μM) of PPIX for 48 h, cell viability was determined using MTT assay. **(C)** SK-MEL-2 and HNM were treated with PPIX (10, 20, and 30 μM) for 48 h, and melanin contents were measured. **(D)** SK-MEL-2 cells were treated with PPIX for 48 h and then stained with Masson-Fontana ammoniacal silver stain. Bar = 20 μm. Total length of dendrites per cell was measured on the pictures using ruler. Data are expressed as the mean ± SEM (n = 3). *p < 0.05, **p < 0.01, ***p < 0.001 versus non-treated cells.

PPIX is a heterocyclic organic compound that consists of four pyrrole rings (**Figure 1A**). Large amounts of PPIX produce skin damage (Chen et al., 2002; Dailey and Meissner, 2013). A cell viability assay was first performed to measure the levels of PPIX cytotoxicity. No cytotoxic effects were observed at concentrations below 30 μM after 48 h of exposure to PPIX (**Figure 1B**). Then, we chose 10, 20, and 30 μM as the optimal concentrations to measure the effect of PPIX on melanogenesis. As shown in **Figure 1C**, PPIX significantly promoted melanin synthesis in SK-MEL-2 cells and human normal melanocytes (HNM). Furthermore, the Fontana-Masson staining indicated that PPIX increased the number and length of dendrites and melanin concentration in dendrites in SK-MEL-2 cells (**Figure 1D**). These effects were similar to those of positive drug α-MSH (**Figure S1**).

### PPIX Increased Cellular Tyrosinase Activity and the Expression Levels of Tyrosinase, TRP-1, TRP-2, and MITF

**Figure 2:**
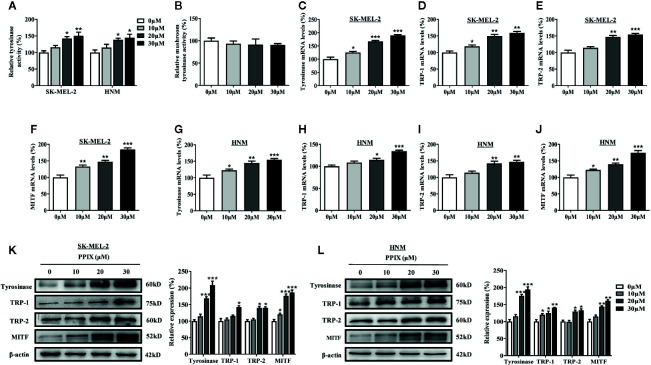
PPIX increased the tyrosinase activity and expression of tyrosinase and MITF. **(A)** Cellular tyrosinase activity and **(B)** mushroom tyrosinase activity in a cell-free assay system were measured after PPIX-treated as described in methods. SK-MEL-2 and HNM cells were treated with PPIX (10, 20, and 30 μM) for 12 h, and RT-qPCR was then applied to detect tyrosinase **(C**, **G)**, TRP-1 **(D**, **H)**, TRP-2 **(E**, **I)**, and MITF **(F**, **J)** gene expression. **(K)** SK-MEL-2 cells and **(L)** HNM were treated with PPIX (10, 20, and 30 μM) for 48 h, and western blot was then applied to detect the protein levels of tyrosinase, TRP-1, TRP-2, and MITF. Data are expressed as the mean ± SEM (n = 3). *p < 0.05, **p < 0.01, ***p < 0.001 versus non-treated cells.

Tyrosinase plays an important role in melanogenesis (Wang et al., 2017). MITF is a key activator of the tyrosinase promoter (Lee et al., 2013; Lv et al., 2020). First, we measured the effects of PPIX on cellular and cell-free tyrosinase activity. As shown in **Figure 2A**, PPIX treatment at doses ranging from 10 to 30 μM led to a dose-dependent increase in cellular tyrosinase activity in SK-MEL-2 and HNM cells. However, PPIX did not directly affect the enzymatic activities of tyrosinase (**Figure 2B**). To understand the underlying mechanism by which PPIX regulates melanogenesis, we evaluated the mRNA and protein levels of melanogenesis-related genes in melanocytes by RT-PCR and WB. As shown in **Figures 2C–J**, PPIX increased the mRNA levels of tyrosinase, TRP-1, TRP-2, and MITF in SK-MEL-2 and HNM cells. Western blot analysis indicated that PPIX upregulated the expression levels of tyrosinase, TRP-1, TRP-2, and MITF in SK-MEL-2 and HNM cells (**Figures 2K, L**). These findings suggest that PPIX induces melanogenesis by upregulating the expression of the related melanogenesis proteins.

### PPIX Regulates Melanosome Transport

**Figure 3:**
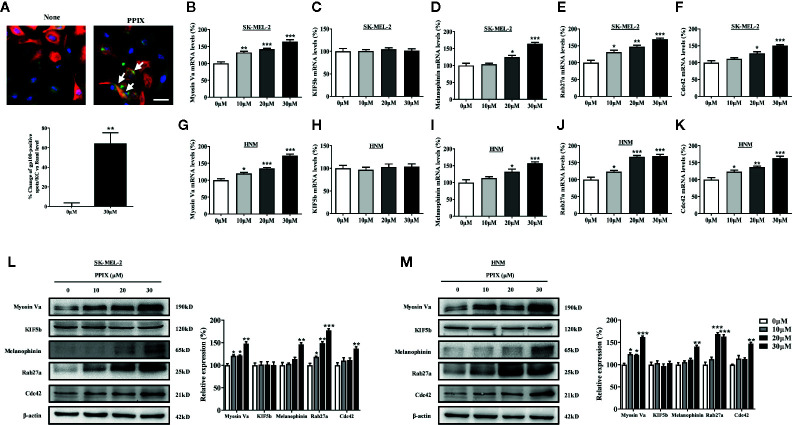
PPIX induced melanosome transport in melanocytes. **(A)** In cocultured SK-MEL-2 and HaCaT cells, melanosome with yellow signal (arrow) were evident in cytokeratin-positive HaCaT cells. Melanosome labeling with GP100 (green). HaCaT cells labeling with cytokeratin (red). Bar = 20 μm. Quantification of melanosomes transferred to HaCaT cells. Twenty cells per condition were assessed in each of three independent experiments. SK-MEL-2 cells and HNM were treated with PPIX (10, 20, and 30 μM) for 12 hours, and RT-qPCR was then applied to detect myosin Va **(B**, **G)**, KIF5b **(C**, **H)**, melanophinin **(D**, **I)**, Rab27a **(E**, **J)**, and Cdc42 **(F**, **K)** gene expression. **(L)** SK-MEL-2 and **(M)** HNM cells were treated with PPIX (10, 20, and 30 μM). After 48-h treatment, the expression levels of myosin Va, KIF5b, melanophinin, Rab27a, and Cdc42 were examined. The results were shown as relative values to the control. Data are expressed as the mean ± SEM (n = 3). *p < 0.05, **p < 0.01, ***p < 0.001 versus non-treated cells.

The distribution of melanin in the skin is completed by melanosome transport to the cell membrane and transfer to neighboring keratinocytes (Beaumont et al., 2011; Wu et al., 2018). While KIF5b contributes to outward melanosome transport along microtubules (Hara et al., 2000; Hirokawa et al., 2009), Rab27A-melanophilin-myosin Va complexes regulate melanosome transport along actin filaments (Raposo and Marks, 2007). Rab27a-melanophilin is also responsible for anchoring melanosomes to the cell membrane (Ohbayashi and Fukuda, 2012). Furthermore, Cdc42 contributes to dendrite extension, which is a fundamental requirement for melanosome transfer (Luo, 2000). As shown in **Figure 1D**, melanosomes were produced around the nuclei of SK-MEL-2 cells, whereas PPIX enriched dendrite melanosomes. To further investigate whether PPIX is involved in melanosome transfer to keratinocytes, we examined the effects of PPIX on melanin transfer in co-cultured SK-MEL-2 and HaCaT cells using confocal microscopy. As shown in **Figure 3A**, PPIX increased GP100-positive melanosome transfer in co-cultured SK-MEL-2 and HaCaT cells. These effects were similar to those of positive drug α-MSH (**Figure S2**). To understand the underlying mechanism by which PPIX regulates melanosome transfer, we measured several crucial factors involved in melanosome transport. RT-PCR analysis indicated that PPIX increased the mRNA levels of myosin Va, melanophilin, Rab27a, and Cdc42 in SK-MEL-2 and HNM cells, while the mRNA levels of KIF5b did not significantly change (**Figures 3B–K**). These results were also confirmed in the Western blot analysis (**Figures 3L, M**).

### PPIX Promotes the cGMP/PKG Signaling Pathway by Activating GC

**Figure 4:**
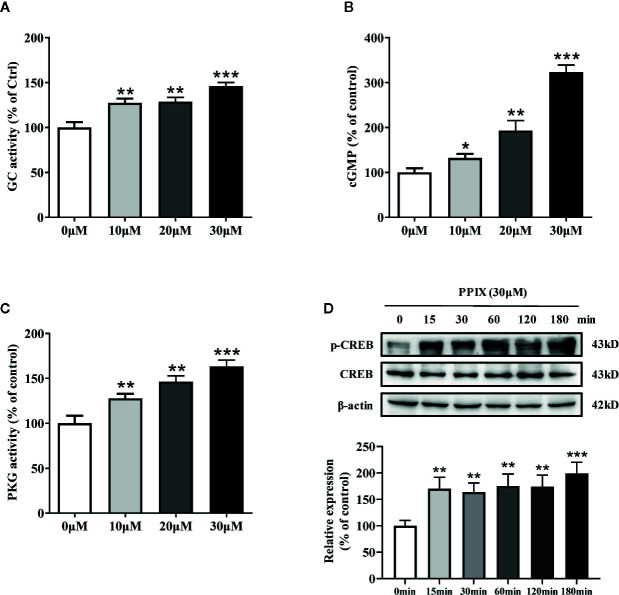
The effects of PPIX on the activity of GC/cGMP/PKG signaling pathways in melanocytes. **(A)** Guanylate cyclase (GC) activity in melanocytes treated with PPIX was measured. Effect of PPIX on cellular cGMP levels **(B)** and PKG activity **(C)** in melanocytes was examined after cells were treated with PPIX. **(D)** Quantification of cAMP-response element-binding protein (CREB) and phosphorylation of CREB (p-CREB) protein expression levels were evaluated by western blotting. Data are expressed as the mean ± SEM (n = 3). *p < 0.05, **p < 0.01, ***p < 0.001 versus non-treated cells.

Previous research has shown that PPIX markedly activates soluble GC purified from bovine lung (Ignarro et al., 1984). GC plays an important role in melanogenesis and UV radiation stimulates pigmentation mainly by activating GC (Liu et al., 2019). After GC is activated, it increases the intracellular cGMP levels and activates PKG/CREB signaling, finally increasing pigmentation (Romero-Graillet et al., 1996). To further clarify the underlying mechanisms of PPIX-induced pigmentation, we measured the relevant signaling pathway activity after PPIX treatment and found that GC activity and cGMP levels markedly increased after PPIX treatment at concentrations of 10, 20, and 30 μM for 10 min (**Figures 4A, B** and **Figure S1**). As shown in **Figure 4C** and **Figure S3**, PPIX increased PKG activity in HNM and SK-MEL-2 cells. Finally, the phosphorylation of CREB was significantly enhanced after PPIX treatment in HNM and SK-MEL-2 cells (**Figure 4D** and **Figure S3**). These results expand upon the results of previous studies and suggest that PPIX directly activates the GC/cGMP/PKG signaling pathway in melanocytes.

PPIX is a heterocyclic organic compound that consists of four pyrrole rings and is the immediate precursor to heme (Sachar et al., 2016). Its tetrapyrrole structure enables it to chelate transition metals to form metalloporphyrins, which execute various biological functions. Previous studies suggested that heme had no effect on GC activity and that an open central core in the porphyrin ring is essential for GC activation (Ignarro et al., 1984). As shown in **Figure S4**, heme did not promote melanogenesis. These results further indicated that PPIX induced pigmentation by directly activating GC.

### Effect of GC and PKG Inhibitors on PPIX-Stimulated Pigmentation

**Figure 5:**
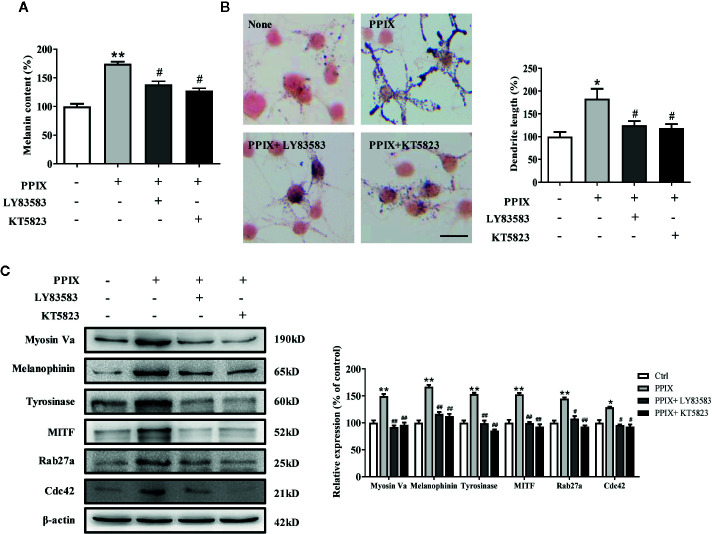
Inhibitory effects of LY83583 and KT5823 on PPIX-induced pigmentation in SK-MEL-2 cells. SK-MEL-2 was pretreated or not with 10 μM LY83583 (or 1 μM KT5823) for 1 h before PPIX was applied for 48 h at 30μM. **(A)** Melanin contents were measured, and **(B)** SK-MEL-2 cells were stained with Masson-Fontana ammoniacal silver stain. Bar = 20 μm. Total length of dendrites per cell was measured on the pictures using ruler. **(C)** Western blotting for the protein expression relating to melanogenesis and melanosome transport were measured. Data are expressed as the mean ± SEM (n = 3). *p < 0.05, **p < 0.01 versus non-treated cells. ^#^p < 0.05, ^##^p < 0.01 versus PPIX-treated cells.

As the GC/cGMP/PKG signaling pathway is activated in melanocytes, we examined whether it is involved in PPIX-induced pigmentation. We incubated melanocytes in the presence of 30 μM PPIX together with 10 μM LY83583 (GC inhibitor) or 1 μM KT 5823 (PKG inhibitor), and then we measured the pigmentation. PPIX-induced stimulation of melanogenesis and dendricity (**Figures 5A, B**) were markedly inhibited by LY83583 and KT5823. In addition, the PPIX-induced increase of tyrosinase, MITF, myosin Va, melanophilin, Rab27a, and Cdc42 expression was blocked by LY83583 or KT5823 (**Figure 5C**), indicating the involvement of GC/cGMP/PKG signaling in PPIX-induced melanosome transport and melanogenesis in melanocytes.

### Confirmation of the Effect of PPIX on Pigmentation in Zebrafish

**Figure 6:**
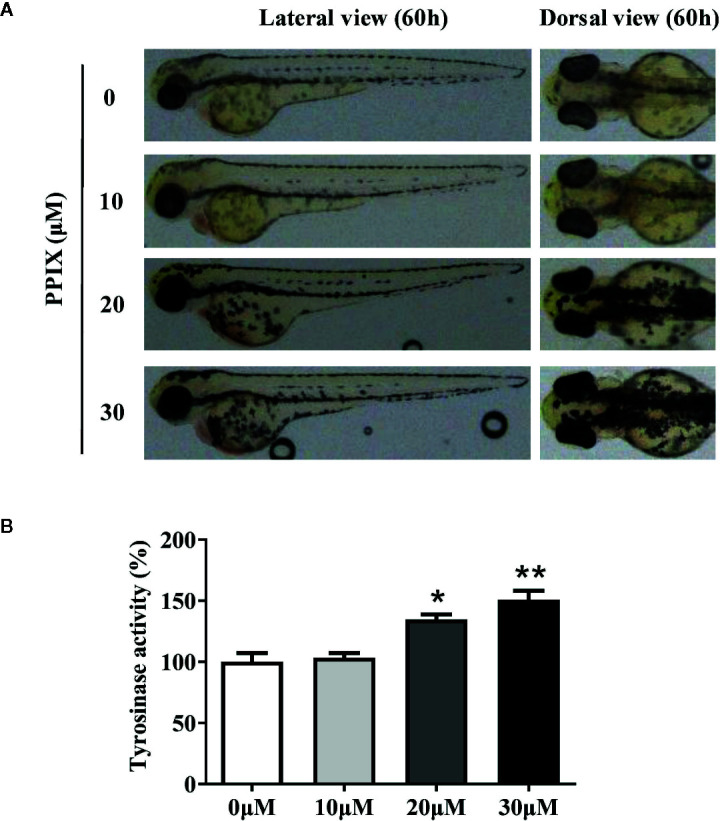
Effect of PPIX on pigmentation in zebrafish. **(A)** Representative photographs of zebrafish. Zebrafish embryos were treated with PPIX from 35 to 60 h. The effects on the pigmentation of zebraﬁsh were observed under the stereomicroscope. **(B)** Tyrosinase activity. For measurement of tyrosinase activity, 100 μg of total protein was incubated with L-DOPA (final, 0.5 mM) and then quantified using a spectrometer. The results were shown as relative values to the control. Data are expressed as the mean ± SEM (n = 3). *p < 0.05, **p < 0.01 versus non-treated zebrafish.

Melanin pigments in zebrafish are seen on the body surface, permitting simple observation of the pigmentation procedure without using complicated experimental processes (Kim et al., 2008). Thus, we confirmed the role of PPIX in melanogenesis in a zebrafish model. As shown in **Figure. 6A**, PPIX significantly stimulated body pigmentation in zebrafish. In addition, PPIX remarkably increased the activity of tyrosinase by utilizing the whole extract of zebrafish (**Figure 6B**).

## Discussion

Numerous research groups are currently concentrating their endeavors to clarify the mechanism and regulation of skin pigmentation to develop new approaches for the treatment of skin pigmentation disorders associated with hyperpigmentation. Despite great efforts, the clinical application of the developed biological and chemical agents is tightly restricted due to their serious adverse cytotoxic effects on skin cells. Therefore, further research is required to reveal the potential mechanism underlying pigmentation and develop safer and more effective melanogenesis-stimulating compounds.

PPIX is a heterocyclic organic compound that consists of four pyrrole rings (Sachar et al., 2016). Accumulation of PPIX produces photosensitivity and skin damage (Thapar and Bonkovsky, 2008; Dailey and Meissner, 2013). In the present study, no cytotoxic effects were detected at concentrations below 30 μM after 48 h of exposure to PPIX. Furthermore, we found that PPIX promoted melanin synthesis in SK-MEL-2 and HNM cells (**Figure 1**), consistent with the results of previous studies (Kim et al., 2006). Tyrosinase activity is critical in the process of melanin synthesis. Although PPIX did not directly affect the enzymatic activities of tyrosinase, it markedly increased the expression levels of tyrosinase, TRP-1, and TRP-2, indicating that PPIX stimulated melanogenesis by promoting the expression of these three pivotal melanin synthesis enzymes (**Figure 2**).

Apart from melanin synthesis, skin pigmentation is determined by various processes, including intracellular melanosome transport and melanosome transfer to adjacent keratinocytes (Beaumont et al., 2011; Wu et al., 2018). Masson–Fontana ammoniacal silver staining and immunofluorescence analysis indicated that PPIX increased melanosome transfer to adjacent keratinocytes, in addition to increasing the number of dendrites and melanosome localization at the cell periphery (**Figures 1D** and **3A**). Furthermore, PPIX increased the expression of Cdc42, which formed dendrites in SK-MEL-2 cells. Melanosome transport is controlled by four critical proteins: KIF5b, myosin Va, melanophilin, and Rab27a. KIF5b functions as a motor regulating outward melanosome transport along microtubules. Following the transport of melanosomes from microtubules to actin filaments, myosin Va-melanophilin-Rab27a complexes contribute to their movement, and Rab27a-melanophilin anchors them to the cell periphery (Chang et al., 2012). Our results revealed that PPIX significantly promoted myosin Va, melanophilin, and Rab27a expression rather than KIF5b expression in SK-MEL-2 and HNM cells (**Figure 3**). These findings suggest that PPIX increases actin-based melanosome transport and localization at the cell periphery by promoting the expression of myosin Va, melanophilin, and Rab27a. However, whether PPIX affects movement of the melanosome along microtubules requires further investigation.

Intracellular cAMP and cGMP are crucial secondary messengers that regulate pigmentation in melanocytes. Adenylate cyclase (AC) promotes intracellular cAMP levels and activates the PKA/CREB pathway (Hunt et al., 1994; Rzepka et al., 2016). Activation of the cAMP/PKA/CREB signaling pathway increases the expression of MITF and tyrosinase, finally promoting melanogenesis. Furthermore, GC increases cGMP levels and, in turn, activates PKG, which promotes melanogenesis *via* activation of the CREB signaling pathway. Moreover, PKG directly activates tyrosinase to increase pigmentation. Several intrinsic and extrinsic compounds, including α-melanocyte stimulating hormone (α-MSH), forskolin, and adrenocorticotropic hormone (ACTH), were reported to promote melanogenesis *via* the cAMP/PKA signaling pathway. However, there are few reports on the compounds activating the cGMP/PKG signaling pathway in melanogenesis. In the present study, PPIX directly activated GC, increased the intracellular levels of cGMP, and subsequently activated the PKG/CREB pathway (**Figure 4** and **Figure S3**). Considering that the GC/cGMP/PKG signaling pathway was activated after PPIX treatment, we investigated whether it was involved in PPIX-induced pigmentation and the upregulation of tyrosinase, MITF, myosin Va, melanophilin, Rab27a, and Cdc42 expression. The GC inhibitor LY83583 and PKG inhibitor KT5823 markedly reversed PPIX-induced melanin synthesis (**Figures 5A, B**) and decreased the expression of tyrosinase, MITF, myosin Va, melanophilin, Rab27, and Cdc42 in melanocytes (**Figure 5C**). These results suggested that PPIX increased melanogenesis by activating GC, which subsequently increased the cGMP levels and PKG activity, finally leading to pigmentation.

Chelation of PPIX with iron forms heme (iron PPIX) (Sachar et al., 2016). PPIX is the final intermediate in the heme biosynthetic pathway. Previous studies suggested that heme had no effect on GC activity and that an open central core in the porphyrin ring is essential for GC activation (Ignarro et al., 1984). As shown in **Figure S4**, heme did not promote melanogenesis. This result further indicated that PPIX induced pigmentation by directly activating GC. Thus, further investigations are required to elucidate the molecular mechanisms underlying how PPIX regulates GC activation.

Finally, we confirmed the role of PPIX in pigmentation in zebrafish, an increasingly attractive and highly advantageous vertebrate model organism, sharing a high genetic and organ system similarity with humans (Kim et al., 2008). Furthermore, zebrafish have melanin pigments on the surface of the body, which permits simple observation without the use of complicated experimental procedures. In the current study, PPIX increased body pigmentation in addition to tyrosinase activity (**Figure 6**). The consistency between the *in vivo* and *in vitro* results is one of the interesting findings of this study. PPIX effectively stimulated pigmentation in zebrafish *in vivo* and effective melanogenesis in skin cells *in vitro*.

**Figure 7:**
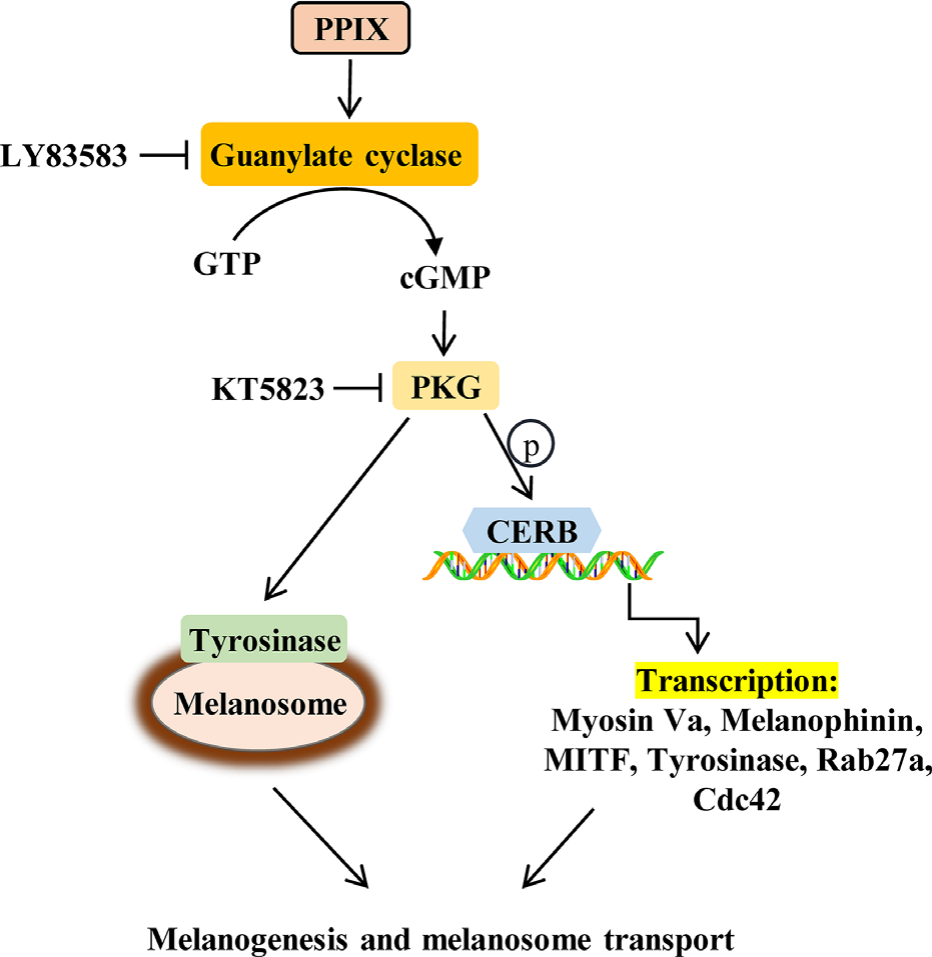
Schematic description of changes in pigmentation upon PPIX treatment.

Overall, our results suggest that PPIX, in addition to its role in heme biosynthesis and photodynamic therapy in cancer, also regulates the processes of melanogenesis and melanosome transport. Furthermore, this function can be ascribed to the activation of the GC/cGMP/PKG signaling pathway, which leads to increased expression of tyrosinase, MITF, myosin Va, melanophilin, Rab27a, and Cdc42, finally promoting melanin synthesis and melanosome transport (**Figure 7**). During the *in vivo* experiments, PPIX induced tyrosinase activity and body pigmentation in zebrafish. Considering that PPIX did not show cytotoxic effects in the present study, it may be utilized as an effective and safe pigmentation enhancer.

## Data Availability Statement

All datasets presented in this study are included in the article/**Supplementary Material**.

## Ethics Statement

The animal study was reviewed and approved by the animal care and use committee of Changzhou University.

## Author Contributions

JL and RG conceived and designed the study, provided critical comments and edited the manuscripts. JL, XA, and SJ carried out major experiments. SJ and YY performed analysis and interpretation of data on immunoblot analysis and Elisa kit assay. GS performed on data collecting. All authors contributed to the article and approved the submitted version.

## Funding

This study was sponsored by the Fund of Changzhou Sci&Tech Program (grant no. CJ20180007) to JL. We would like to thank Editage (www.editage.com) for English language editing.

## Conflict of Interest

Author XA was employed by the company Shanghai Jiyan Biopharmaceutical Co., Ltd.

The remaining authors declare that the research was conducted in the absence of any commercial or financial relationships that could be construed as a potential conflict of interest.
