# Supplementary material for: Protoporphyrin IX Stimulates Melanogenesis, Melanocyte Dendricity, and Melanosome Transport Through the cGMP/PKG Pathway
Source: Front Pharmacol. 2020 Sep 11;11:569368. doi: 10.3389/fphar.2020.569368 (PMC7516199; doi:10.3389/fphar.2020.569368)
Supplement: Supplementary file 1 [file DataSheet_1.docx]

**Table Ⅰ.** Primer sequence (Mouse)

| Genes | Sense (5’-3’) | Antisense (5’-3’) |
| --- | --- | --- |
| GAPDH | AGGTCGGTGTGAACGGATTTG | TGTAGACCATGTAGTTGAGGTCA |
| Tyrosinase | CTCTGGGCTTAGCAGTAGGC | GCAAGCTGTGGTAGTCGTCT |
| TRP-1 | CCCCTAGCCTATATCTCCCTTTT | TACCATCGTGGGGATAATGGC |
| TRP-2 | TTCTGCTGGGTTGTCTGGG | CACAGATGTTGGTTGCCTCG |
| MITF | ACTTTCCCTTATCCCATCCACC | TGAGATCCAGAGTTGTCGTACA |
| Myosin Va | GAGGAAGTGTGGAAATCGGCA | AGTATGTCAGGGTTCCGTAAGT |
| KIF5b | GCGGAGTGCAACATCAAAGTG | CATAAGGCTTGGACGCGATCA |
| Melanophinin | GTTCAGCGGGACTTTGACCTC | GGCACAGTGAGTCTCATTCAGA |
| Rab27a | TCGGATGGAGATTACGATTACCT | TTTTCCCTGAAATCAATGCCCA |
| Cdc42 | CCCATCGGAATATGTACCAACTG | CCAAGAGTGTATGGCTCTCCAC |

**Table Ⅱ.** Primer sequence (Human)

| Genes | Sense (5’-3’) | Antisense (5’-3’) |
| --- | --- | --- |
| GAPDH | GGAGCGAGATCCCTCCAAAAT | GGCTGTTGTCATACTTCTCATGG |
| Tyrosinase | TGCACAGAGAGACGACTCTTG | GAGCTGATGGTATGCTTTGCTAA |
| TRP-1 | TCTCTGGGCTGTATCTTCTTCC | GTCTGGGCAACACATACCACT |
| TRP-2 | CTTGGGCTGCAAAATCCTGC | CAGCACTCCTTGTTCACTAGG |
| MITF | CTCACAGCGTGTATTTTTCCCA | ACTTTCGGATATAGTCCACGGAT |
| Myosin Va | CAGAGTCCGCTTTATTGATTCCA | ATCACCCATGTTCTGACCACT |
| KIF5b | CTGGCCGAGTGCAACATCA | CGATCACGACCGTGTCTTCT |
| Melanophinin | TGCCCATCTGAACGAGACC | GAGCCGATCTTCACGACTCTG |
| Rab27a | GCTTTGGGAGACTCTGGTGTA | TCAATGCCCACTGTTGTGATAAA |
| Cdc42 | CCATCGGAATATGTACCGACTG | CTCAGCGGTCGTAATCTGTCA |


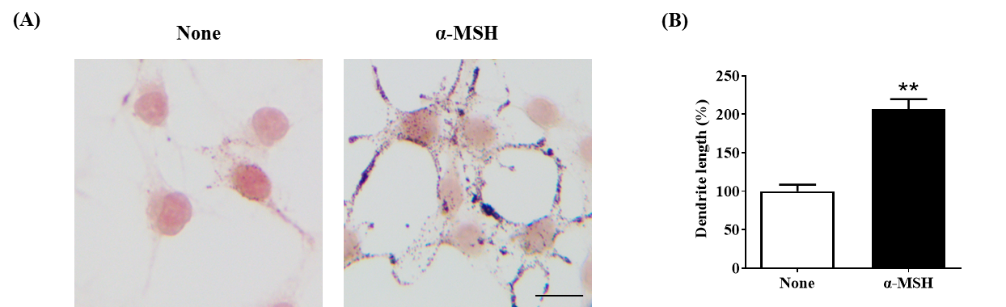


**Figure S1**. α-MSH induced hyperpigmentation in SK-MEL-2 cells. (A) SK-MEL-2 cells were treated with α-MSH (50 nM) for 48 h, and then stained with Masson–Fontana ammoniacal silver stain. Bar=20 μm. (B) Total length of dendrites per cell was measured on the pictures using ruler. Data are expressed as the mean ± SEM (n=3). **p<0.01 versus non-treated cells.


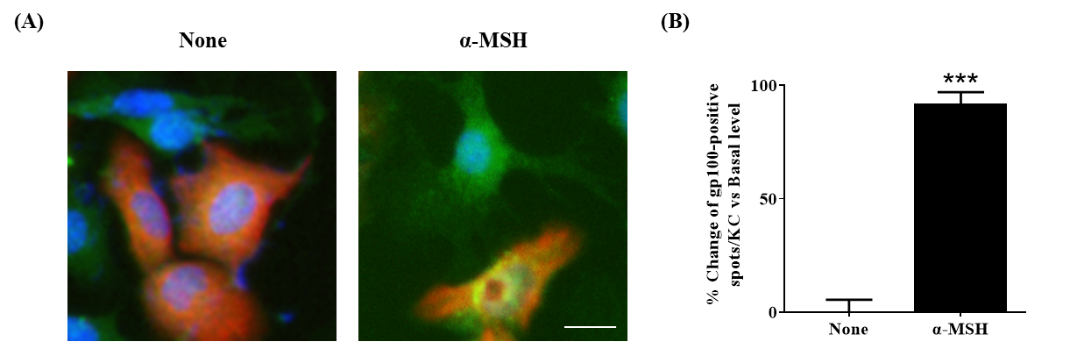


**Figure S2**. α-MSH induced melanosome transport in SK-MEL-2 cells. (A) Cocultured SK-MEL-2 and HaCaT cells were treated with α-MSH (50 nM) for 48 h, and melanosome with yellow signal (arrow) were evident in cytokeratin-positive HaCaT cells. Melanosome labeling with gp100 (green). HaCaT cells labeling with cytokeratin (red). Bar=20 μm. (B) Quantification of melanosomes transferred to HaCaT cells. 20 cells/condition were assessed in each of three independent experiments. Data are expressed as the mean ± SEM (n=3). ***p<0.001 versus non-treated cells.


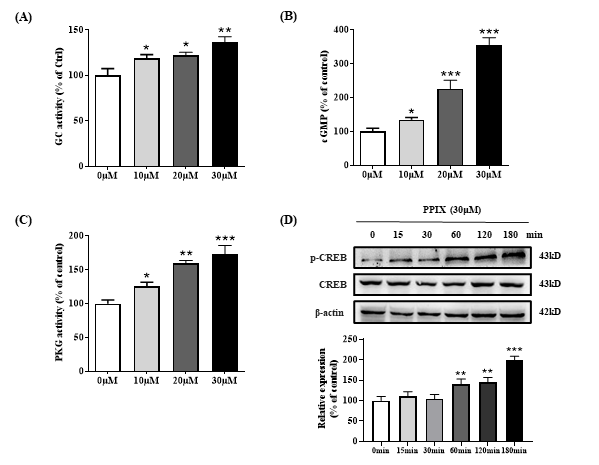


**Figure S3.** The effects of Protoporphyrin IX (PPIX) on the activity of GC/cGMP/PKG signaling pathways in SK-MEL-2 cells. (A) Guanylate cyclase (GC) activity in SK-MEL-2 cells treated with PPIX was measured. Effect of PPIX on cellular cGMP levels (B) and PKG activity (C) in SK-MEL-2 cells was examined after cells were treated with PPIX. (D) Quantification of cAMP-response element-binding protein (CREB) and phosphorylation of CREB (p-CREB) protein expression levels were evaluated by western blotting. Data are expressed as the mean ± SEM (n=3). *p<0.05, **p<0.01, ***p<0.001 versus non-treated cells.

**Figure S4.** The effect of heme on melanogenesis in SK-MEL-2 cells. SK-MEL-2 cells were treated with heme (10, 20, 30 μM) for 48 hours, and melanin contents were measured. Data are expressed as the mean ± SEM (n=3).
