# Supplementary material for: Protoporphyrin IX Stimulates Melanogenesis, Melanocyte Dendricity, and Melanosome Transport Through the cGMP/PKG Pathway
Source: Front Pharmacol. 2020 Sep 11;11:569368. doi: 10.3389/fphar.2020.569368 (PMC7516199; doi:10.3389/fphar.2020.569368)
Supplement: Supplementary file 2 [file DataSheet_2.pdf]

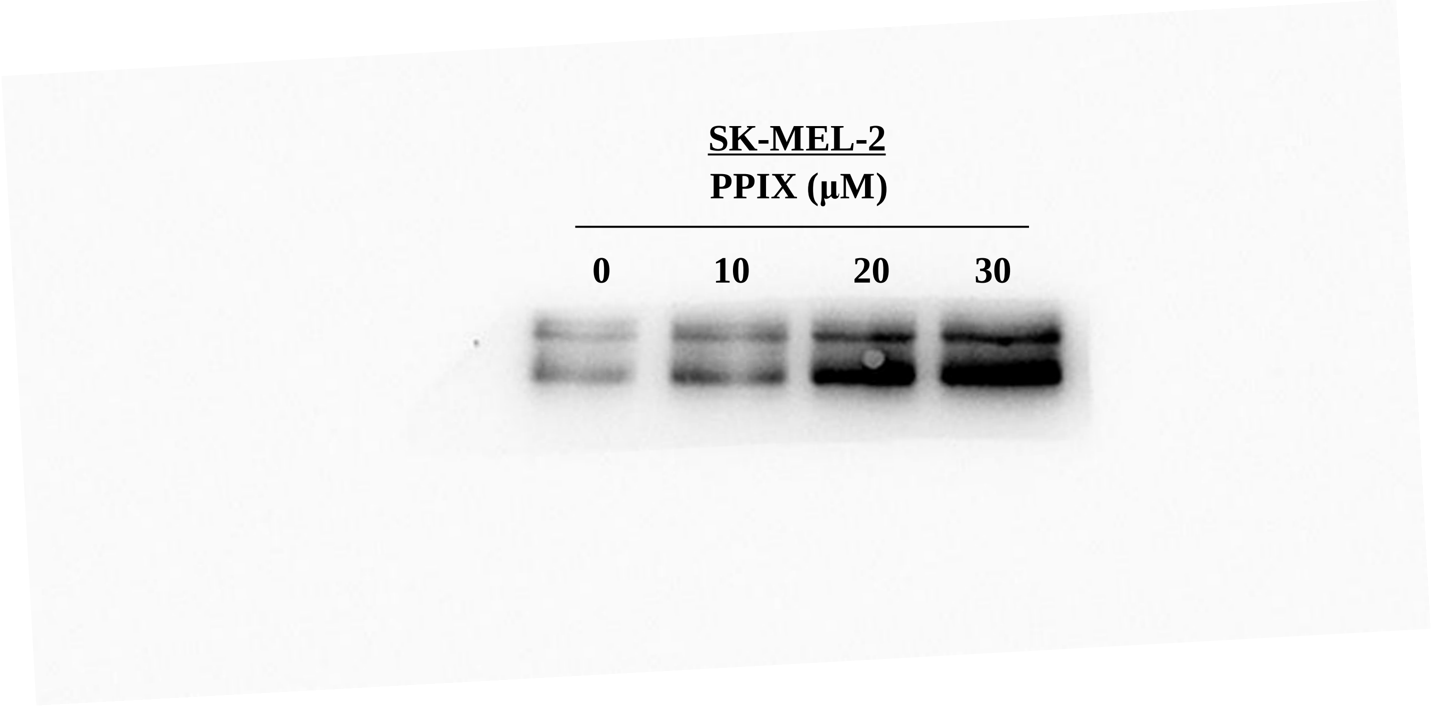

Figure 2C Tyrosinase

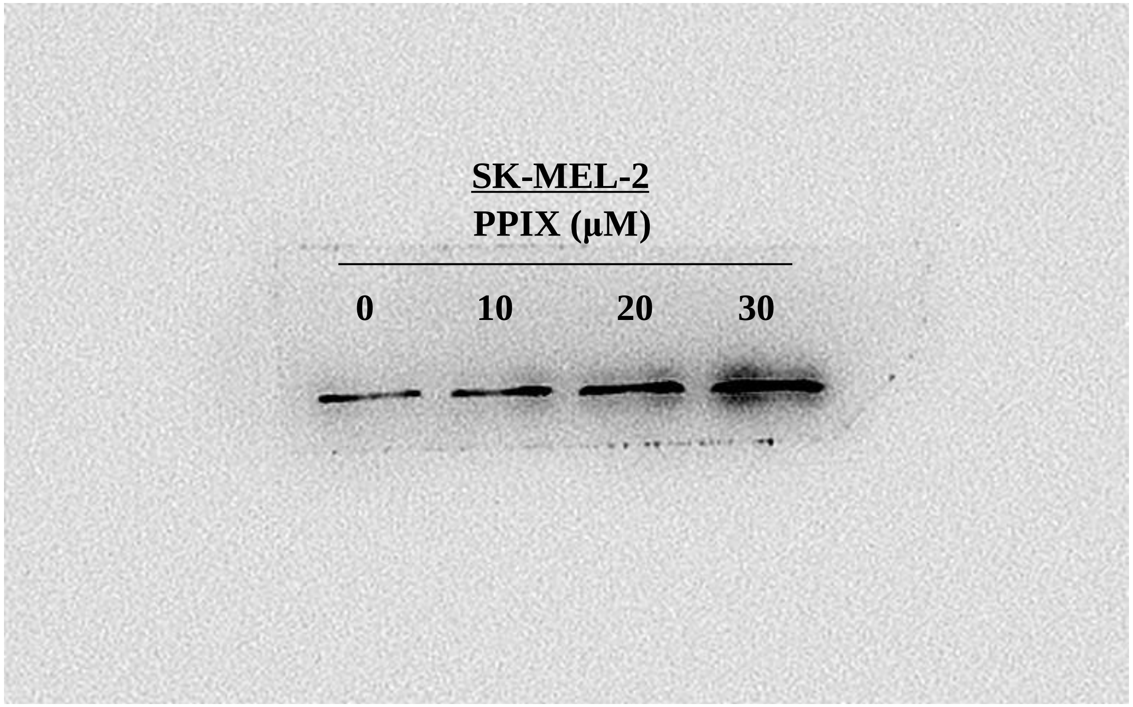

Figure 2C TRP-1

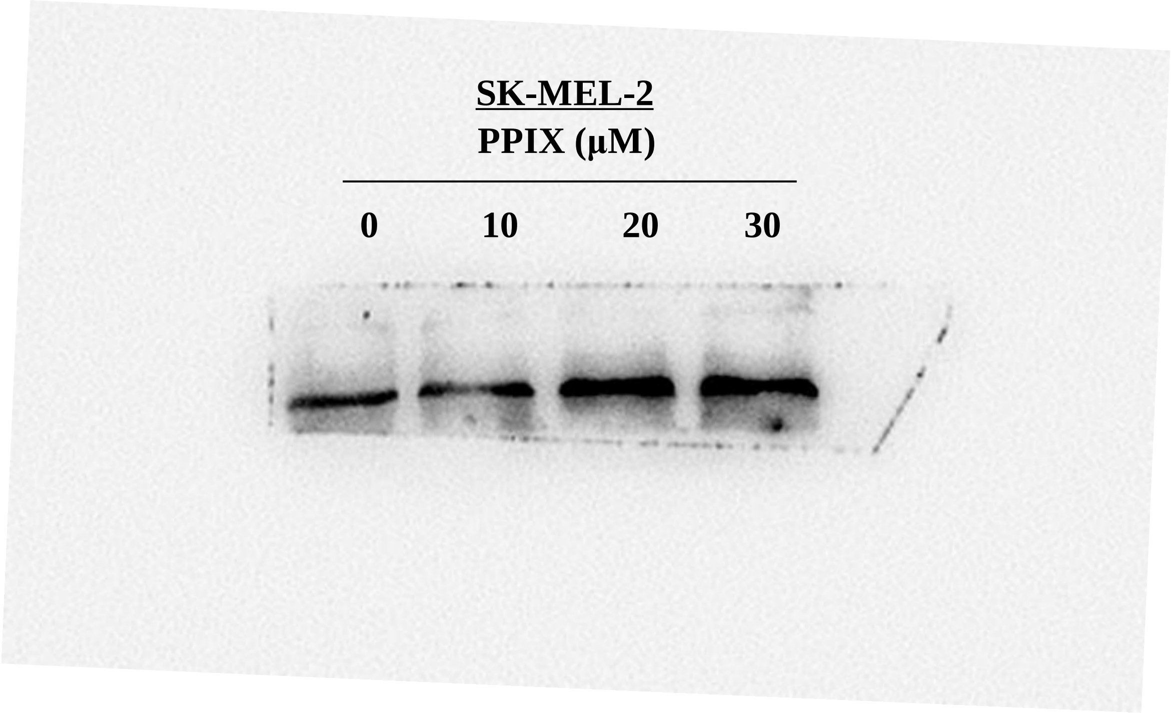

Figure 2C TRP-2

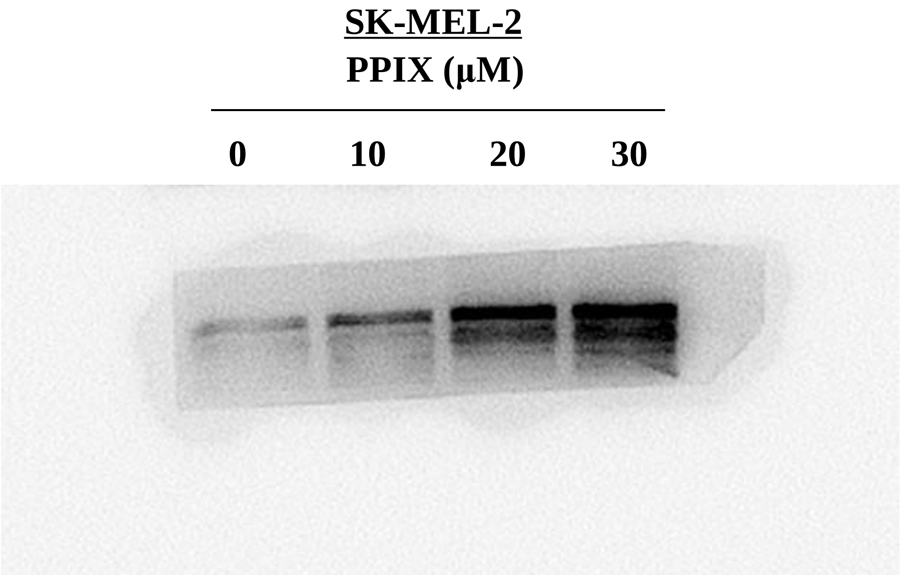

Figure 2C MITF

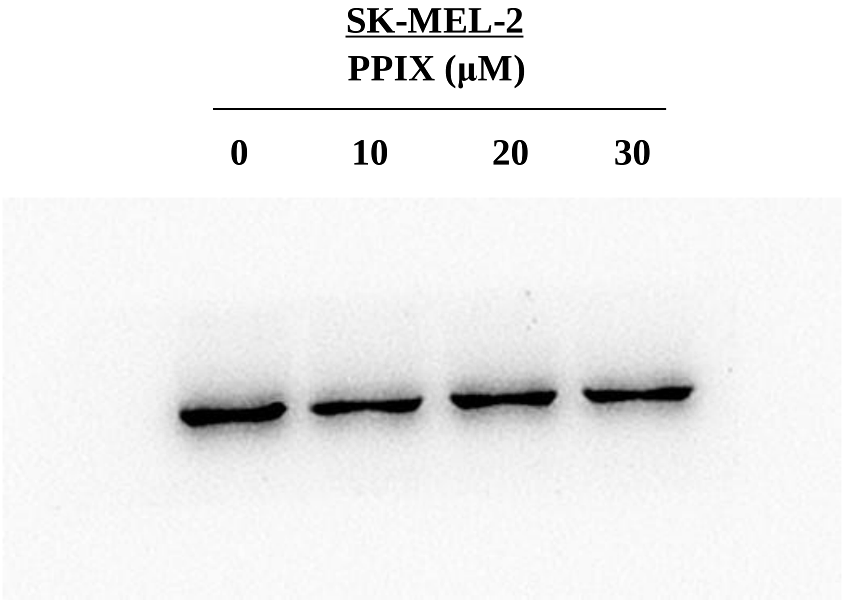

Figure 2C β-actin

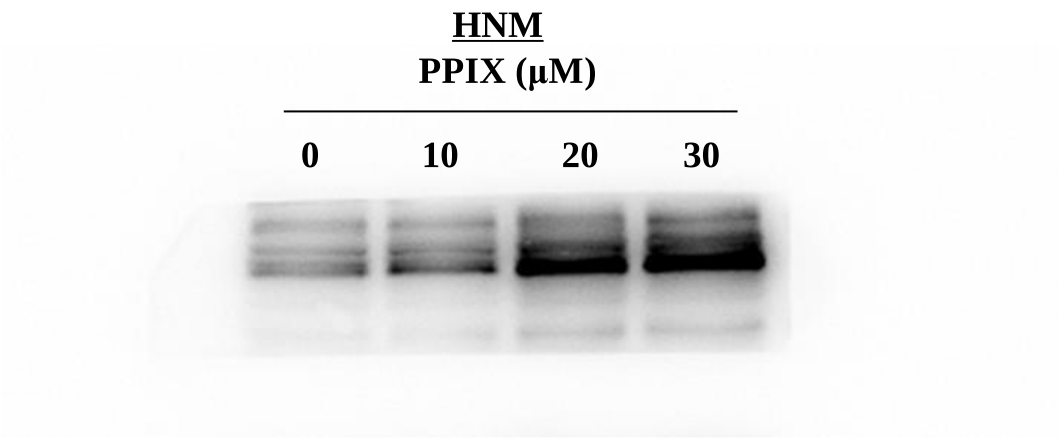

Figure 2D Tyrosinase

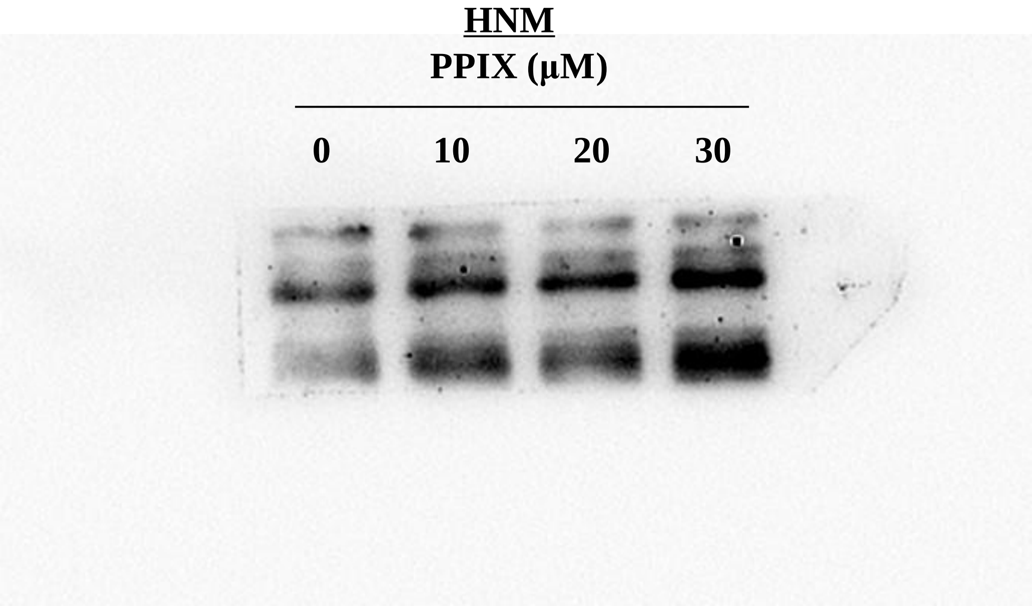

Figure 2D TRP-1

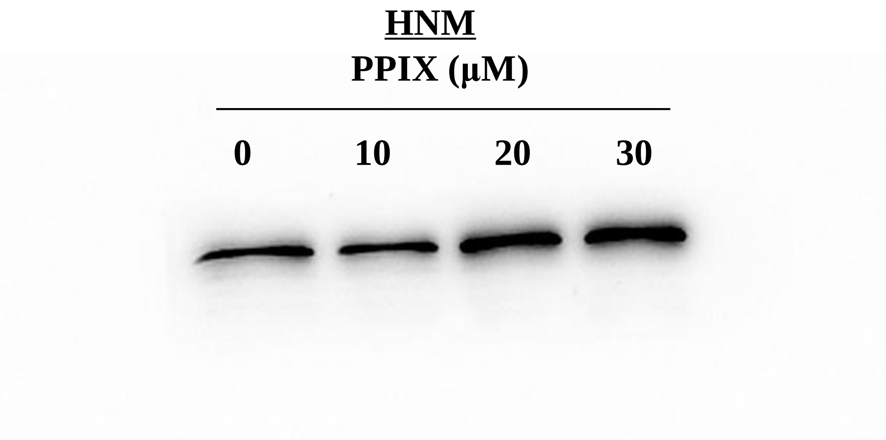

Figure 2D TRP-2

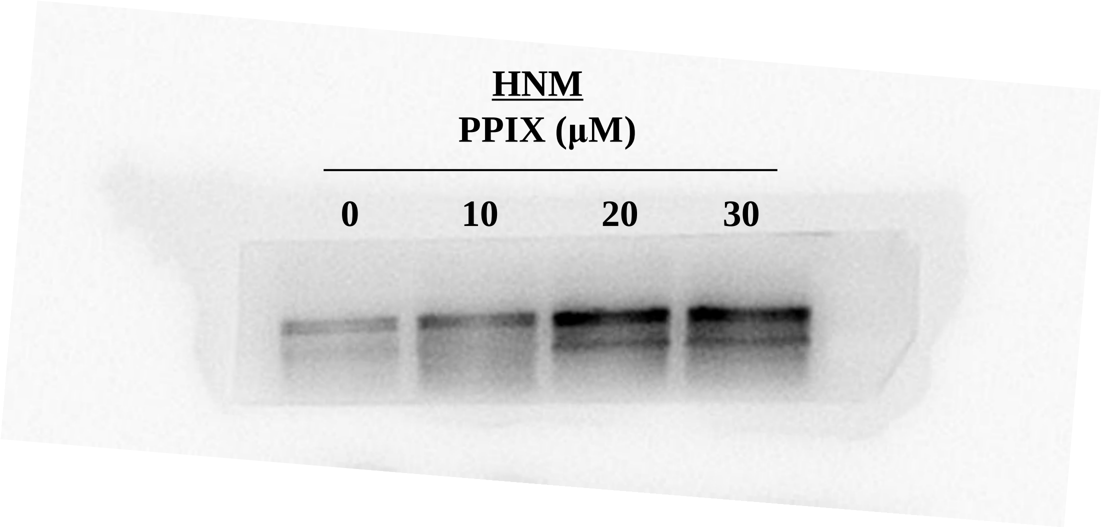

Figure 2D MITF

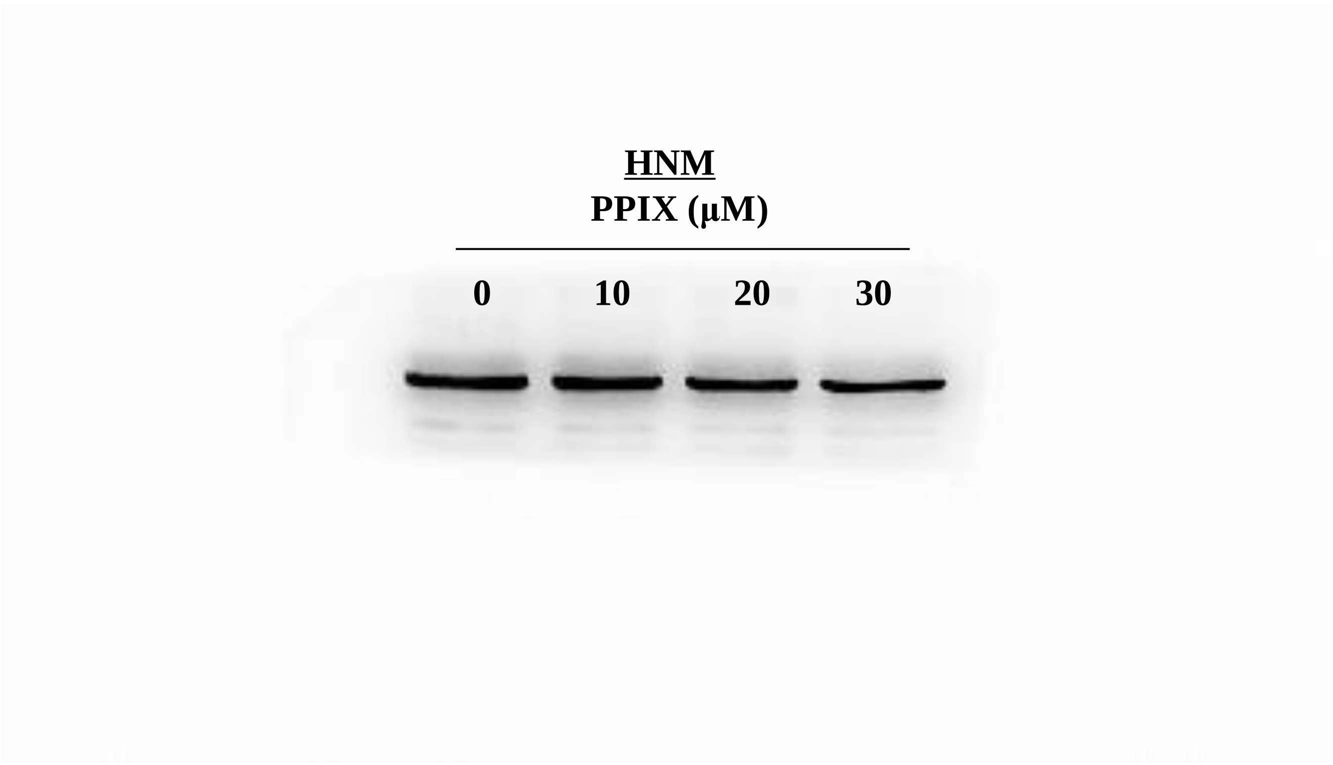

Figure 2D β-actin

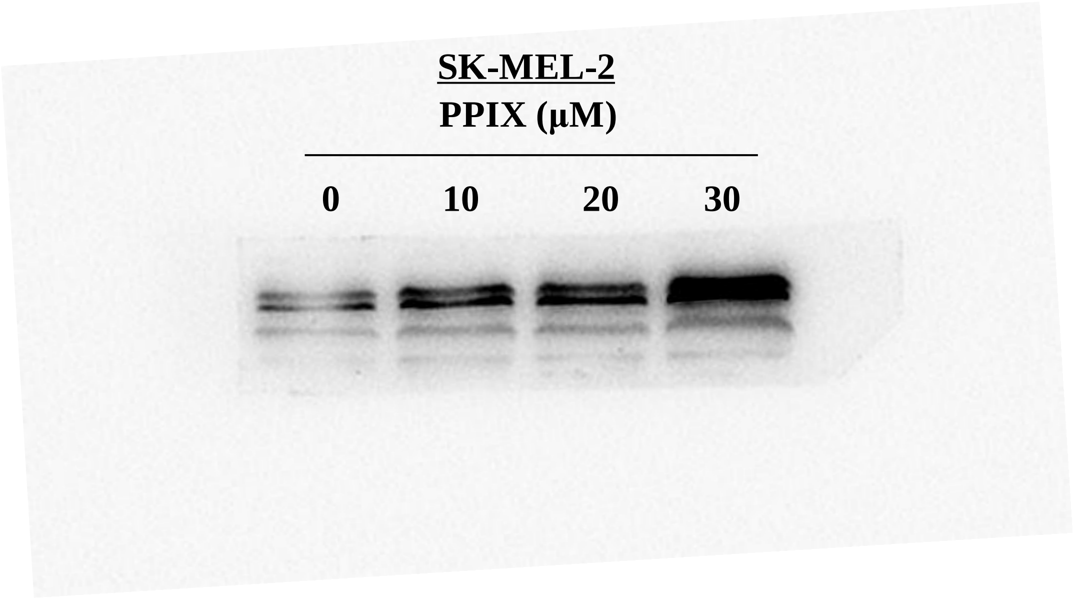

Figure 3B Myosin Va

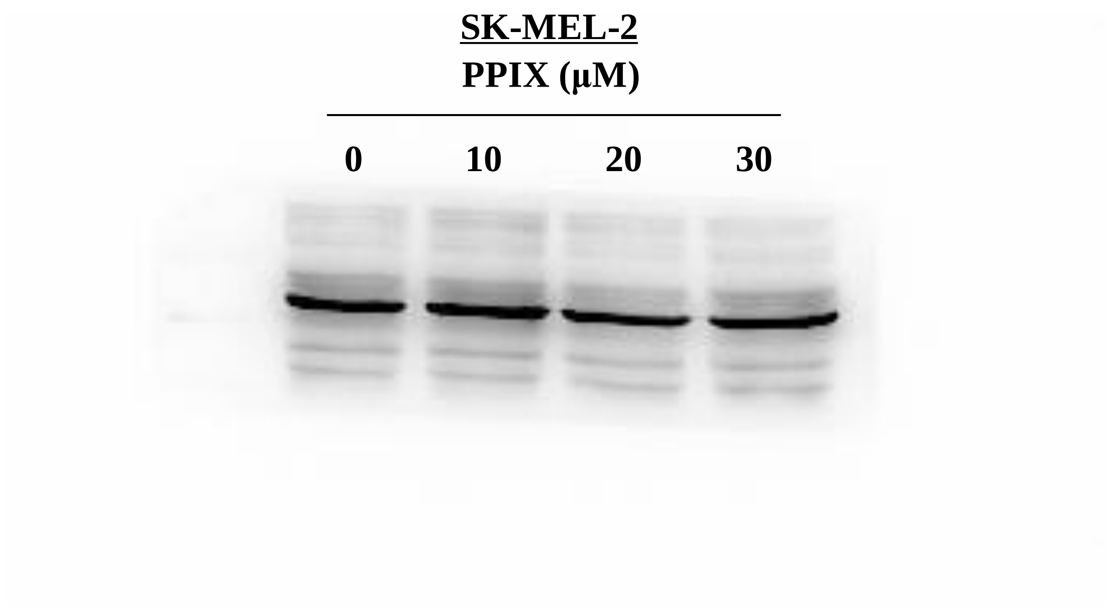

Figure 3B KIF5b

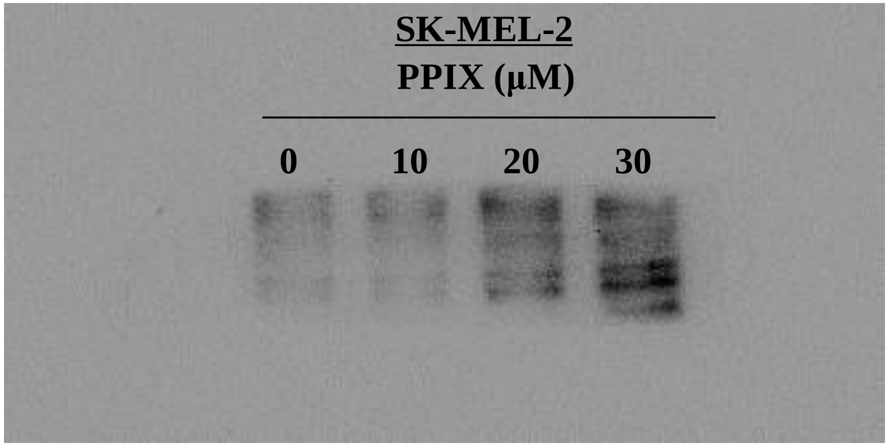

Figure 3B Melanophinin

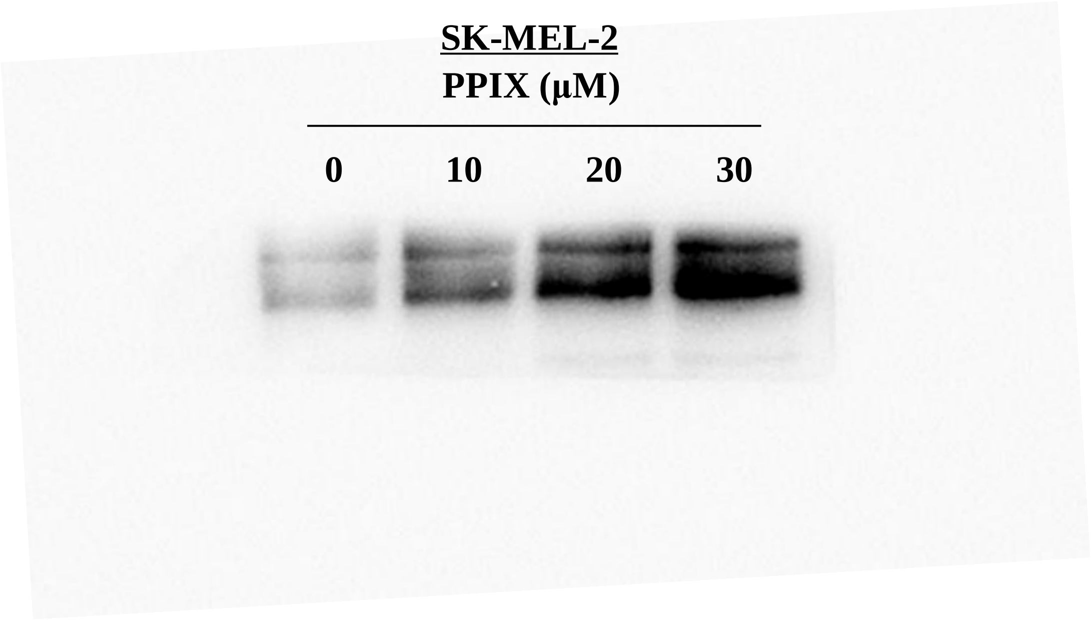

Figure 3B Rab27a

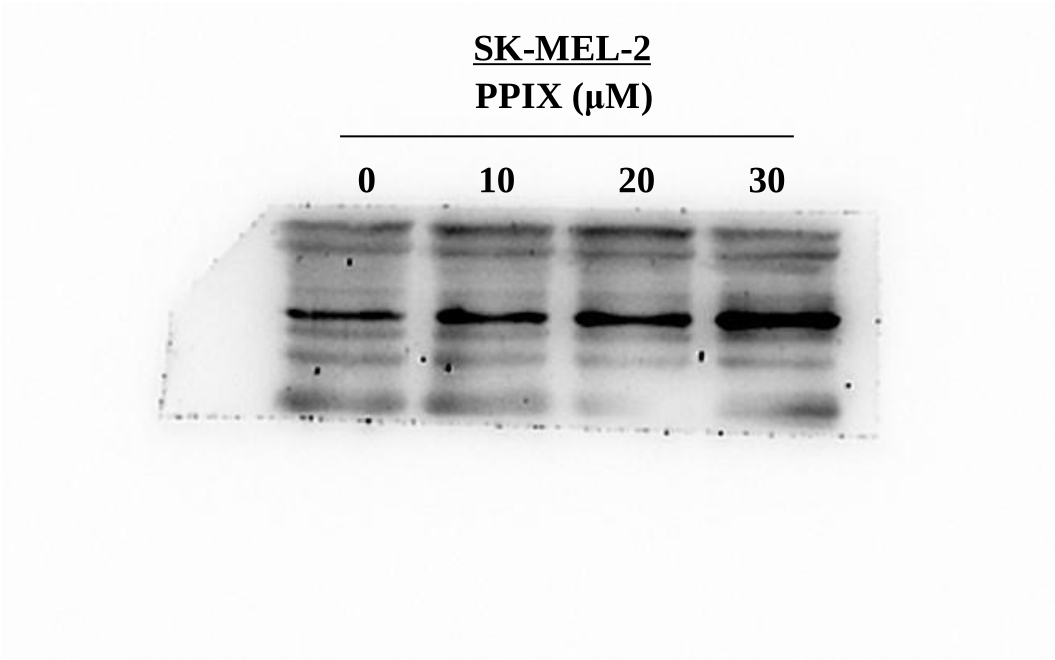

Figure 3B Cdc42

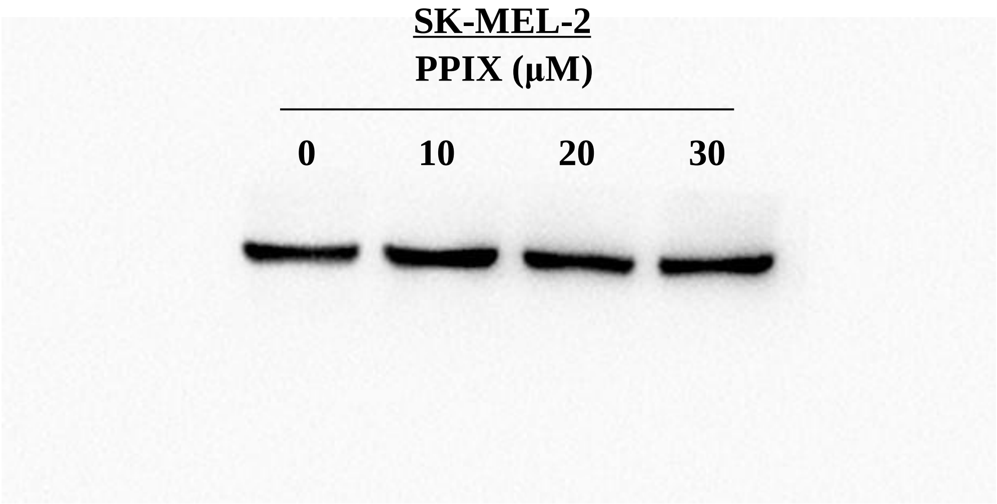

Figure 3B β-actin

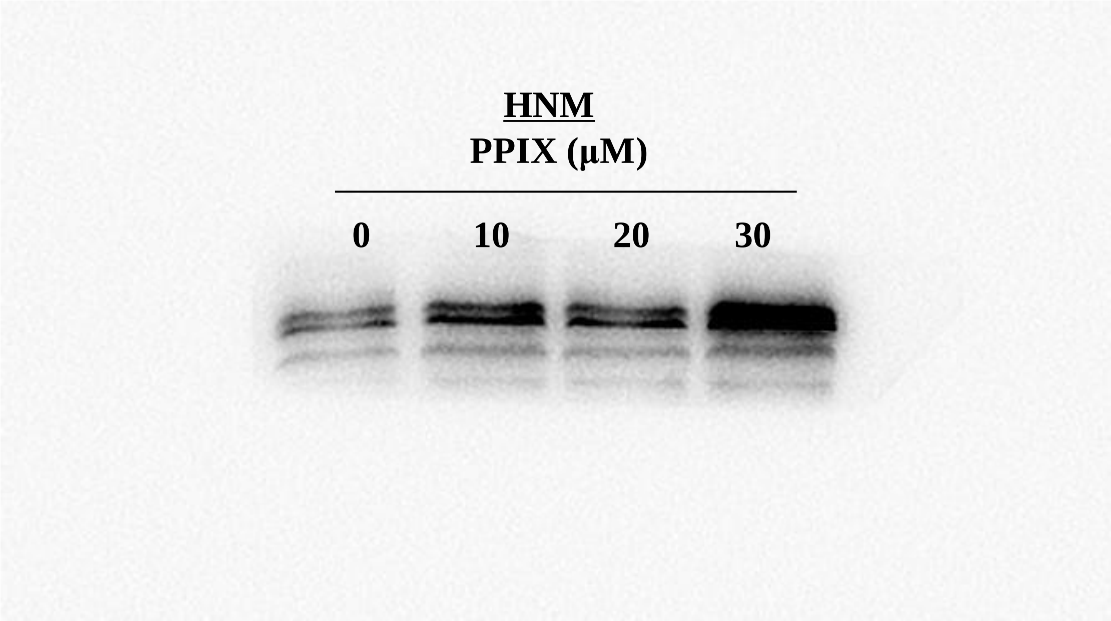

Figure 3C Myosin Va

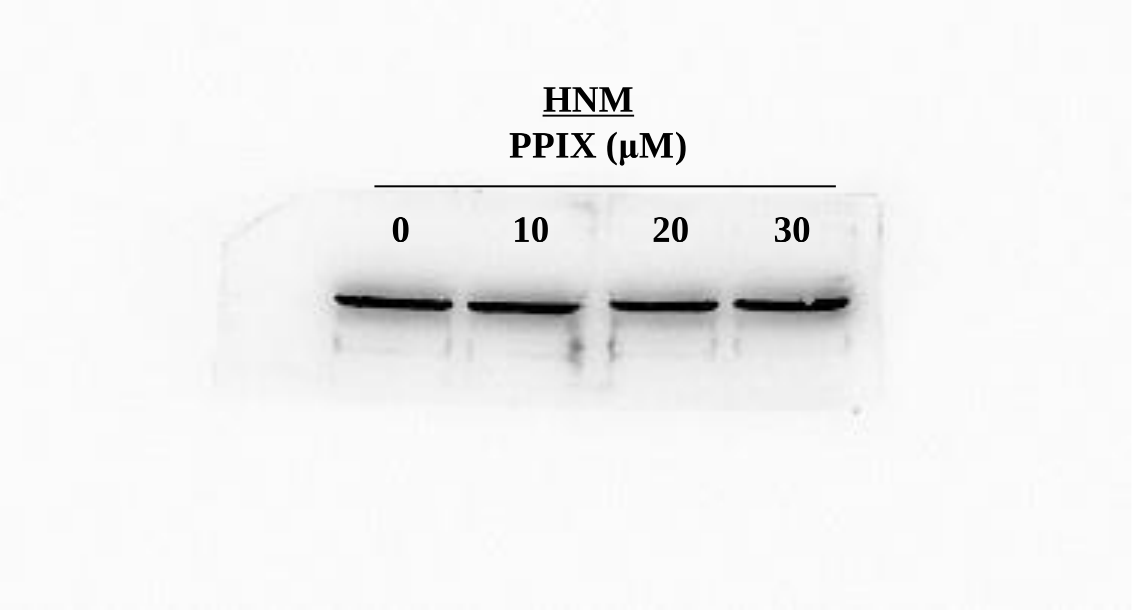

Figure 3C KIF5b

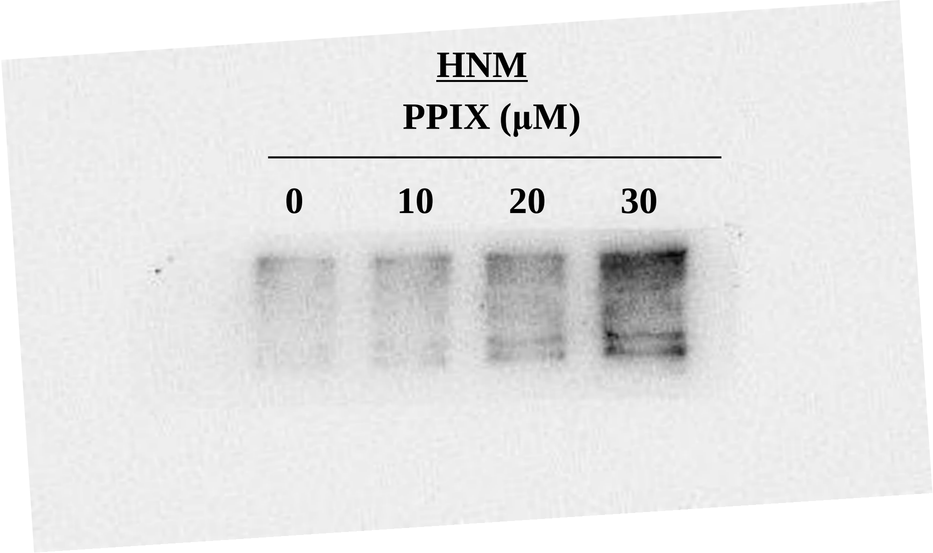

Figure 3C Melanophinin

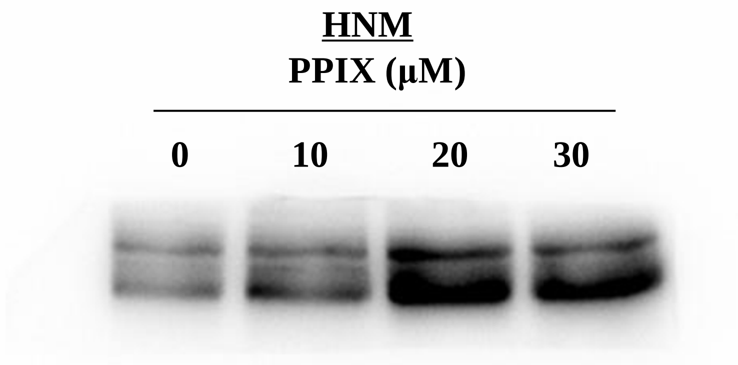

Figure 3C Rab27a

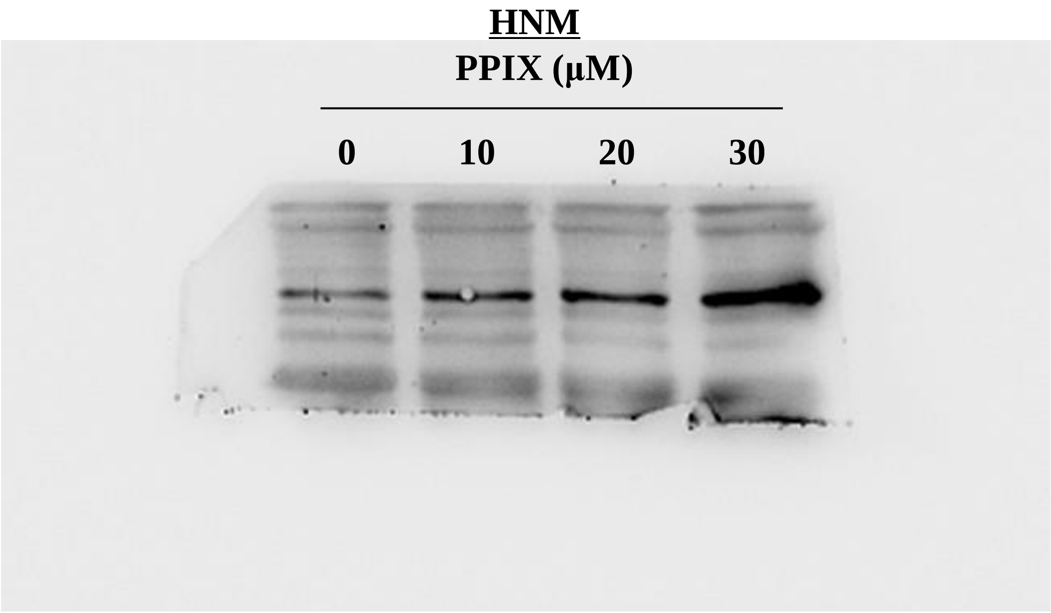

Figure 3C Cdc42

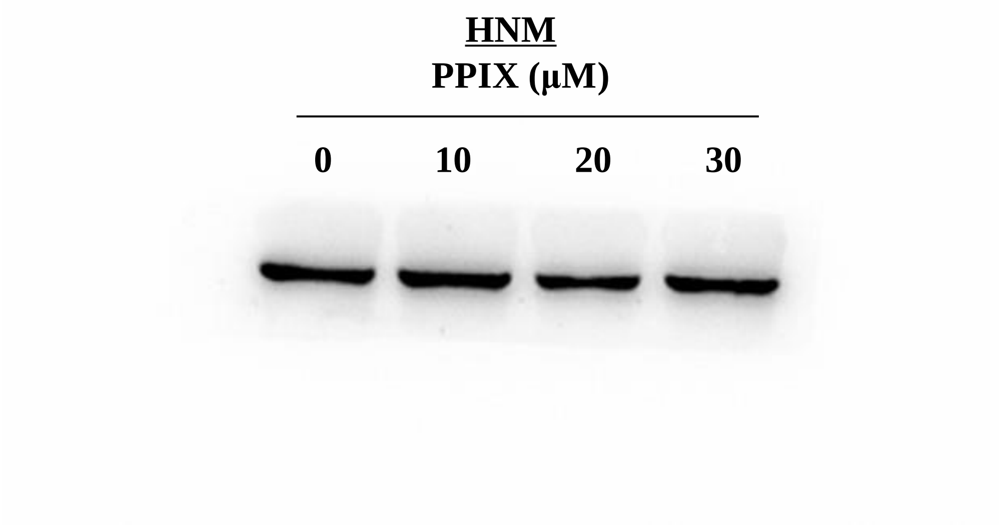

Figure 3C β-actin

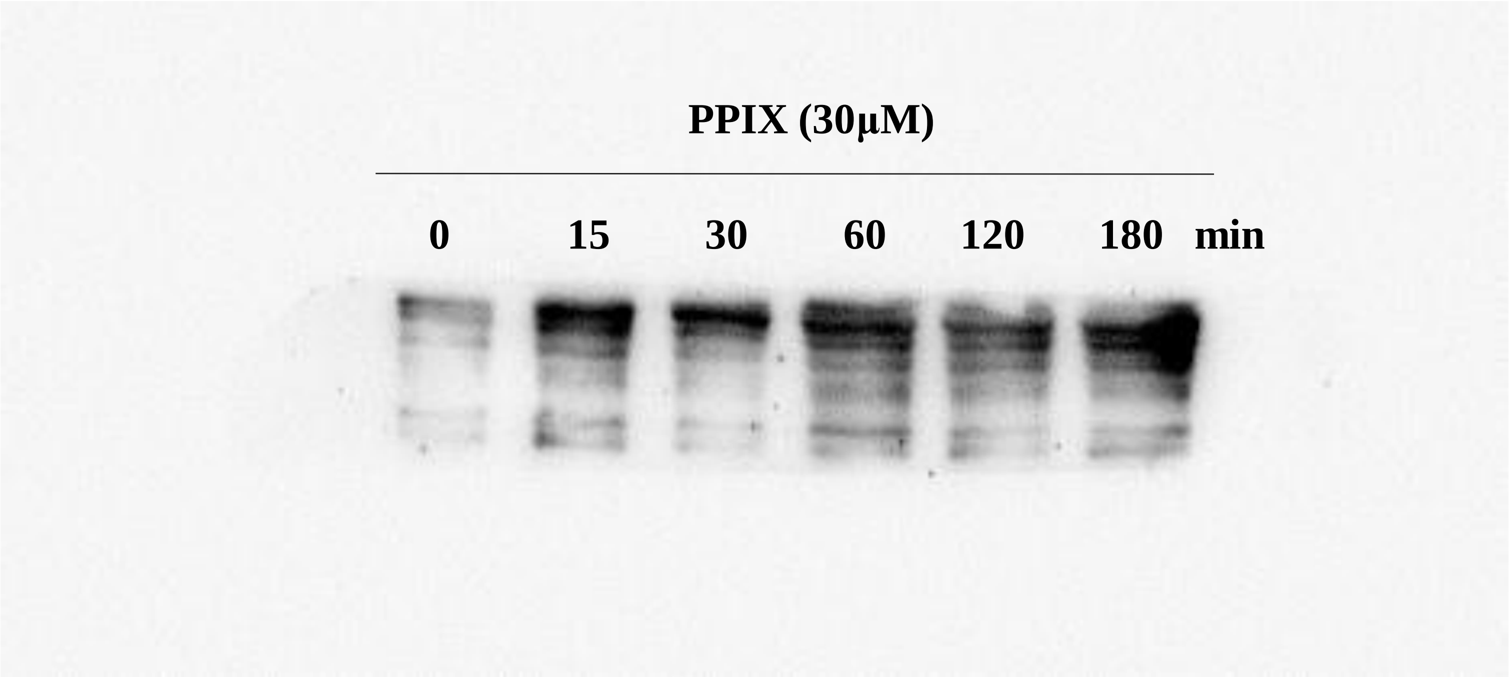

Figure 4E p-CREB

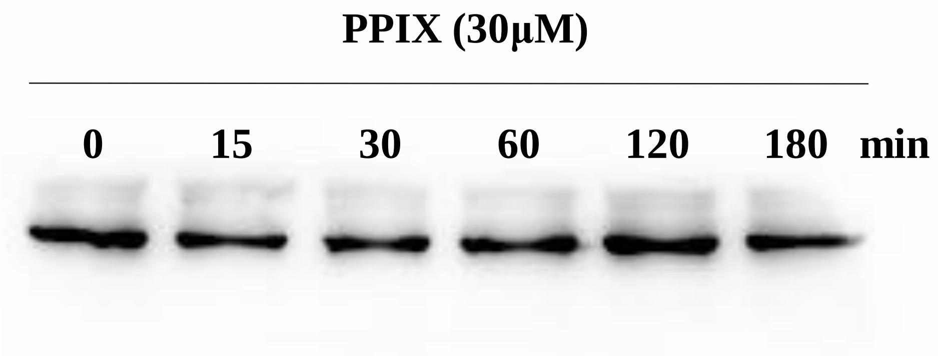

Figure 4E CREB

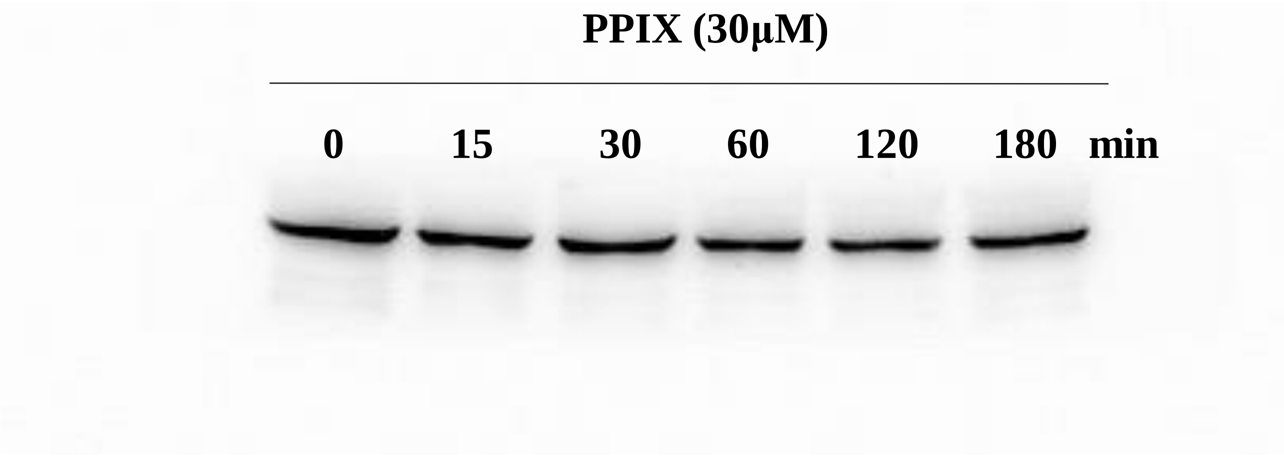

Figure 4E β-actin

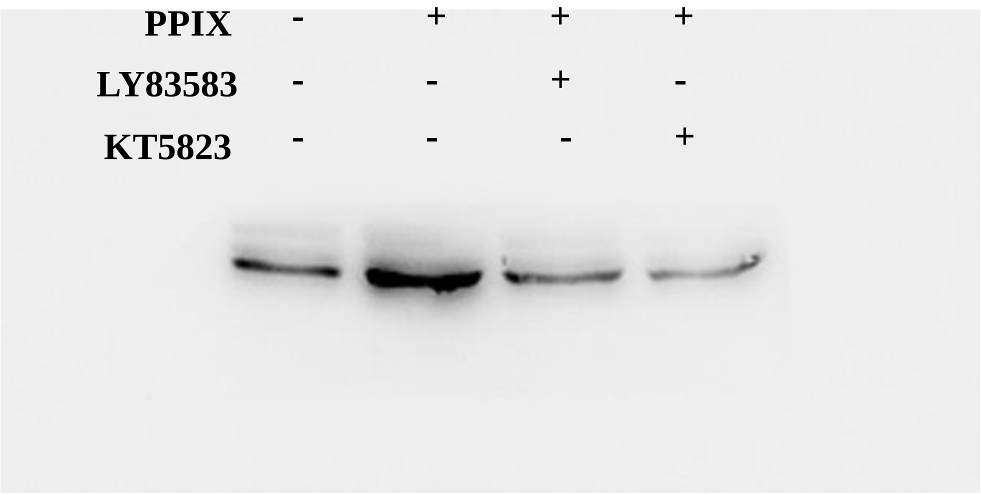

Figure 5C Myosin Va

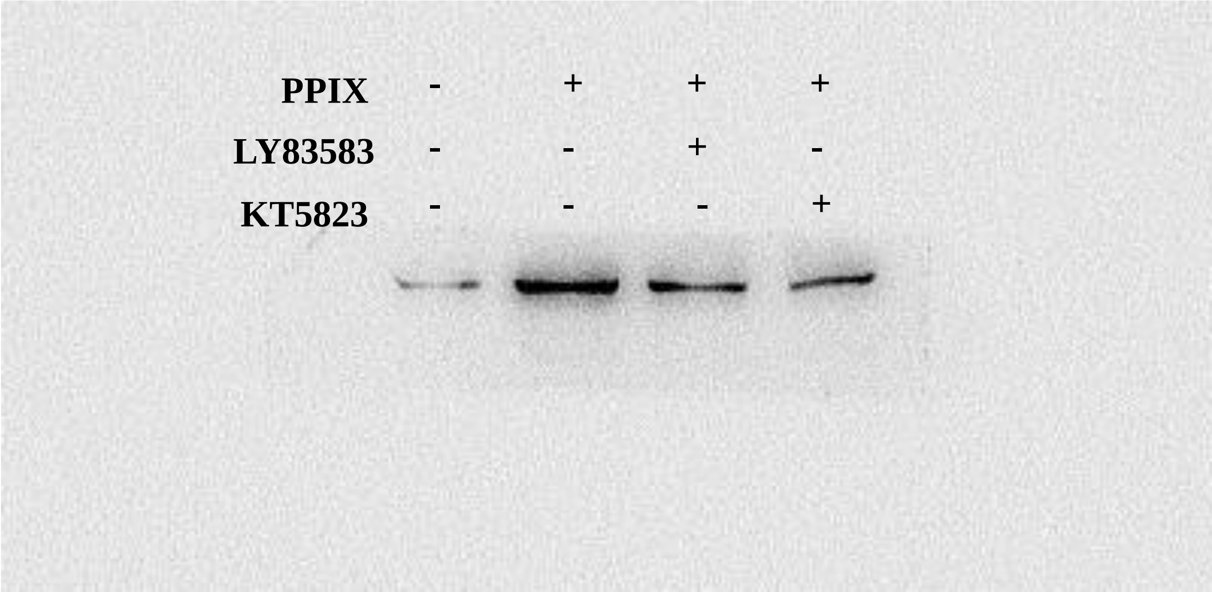

Figure 5C Melanophinin

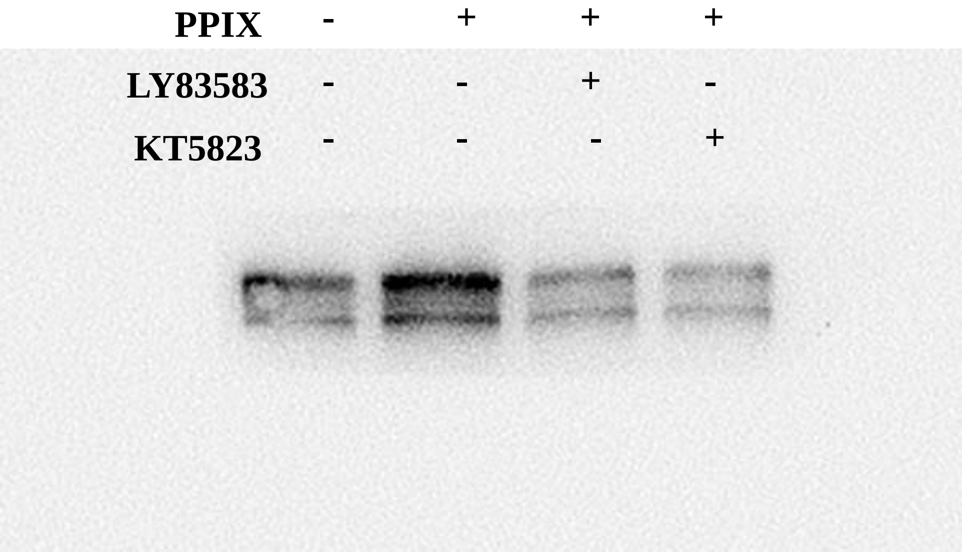

Figure 5C Tyrosinase

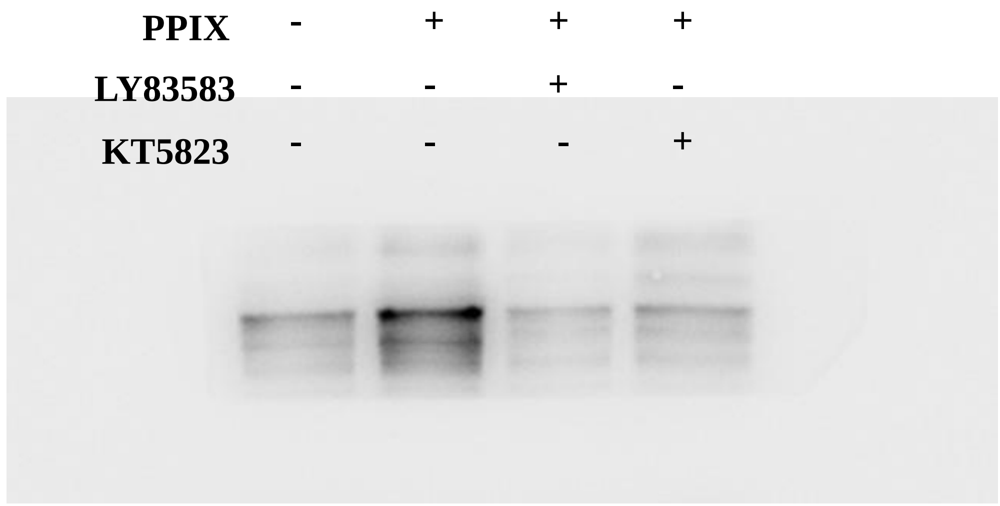

Figure 5C MITF

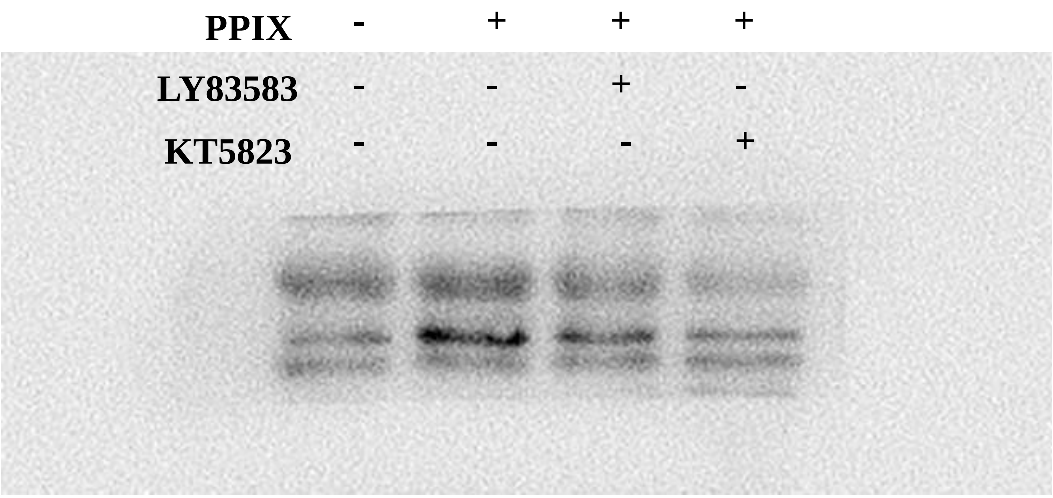

Figure 5C Rab27a

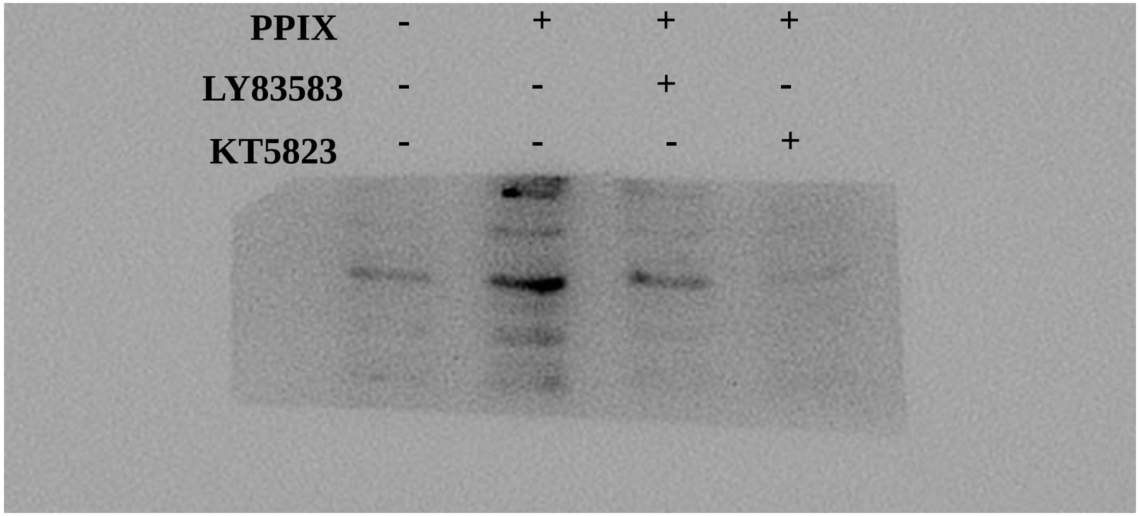

Figure 5C Cdc42

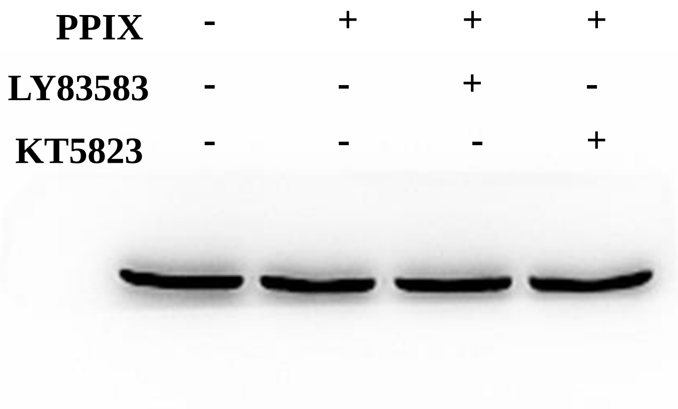

Figure 5C β-actin
